# Advances in brain-targeted delivery strategies and natural product-mediated enhancement of blood–brain barrier permeability

**DOI:** 10.1186/s12951-025-03415-w

**Published:** 2025-05-26

**Authors:** Suyi Liu, Xingyue Jin, Yuanyuan Ge, Junlin Dong, Xinyue Liu, Xiao Pei, Ping Wang, Bing Wang, Yanxu Chang, Xie-an Yu

**Affiliations:** 1 State Key Laboratory of Component-Based Chinese Medicine, Tianjin University of Traditional Chinese Medicine, Tianjin, 301617 China; 2 NMPA Key Laboratory for Bioequivalence Research of Generic Drug Evaluation, Shenzhen Institute for Drug Control, Shenzhen, 518057 China

**Keywords:** Brain diseases, Blood–brain barrier, Natural products, Delivery strategies

## Abstract

The blood–brain barrier (BBB) represents a formidable challenge in the treatment of neurological disorders, as it restricts the passage of most therapeutic agents into the central nervous system (CNS). Research in brain-targeted delivery strategies and explore in natural products for BBB modulation have opened new avenues for effective CNS drug delivery. This review highlights the latest developments in molecular-based delivery systems, cell-based approaches, physical techniques, toxicity concerns, clinical trials and artificial intelligence (AI) -driven modeling for brain-targeted drug delivery. Additionally, it examines the role of natural products, particularly aromatic resuscitation medicines, in enhancing BBB permeability through modulating tight junction proteins and inhibiting efflux transporters. It is emphasized that the integration of natural products with modern drug delivery systems offers promising opportunities for the development of novel brain-targeted therapies. However, challenges related to the complexity and variety of natural product compositions must be addressed to fully realize their potential. This review underscores the importance of continued research into the molecular mechanisms underlying BBB modulation and natural product-mediated nano-delivery strategies for CNS disorders.

**Graphical Abstract:**

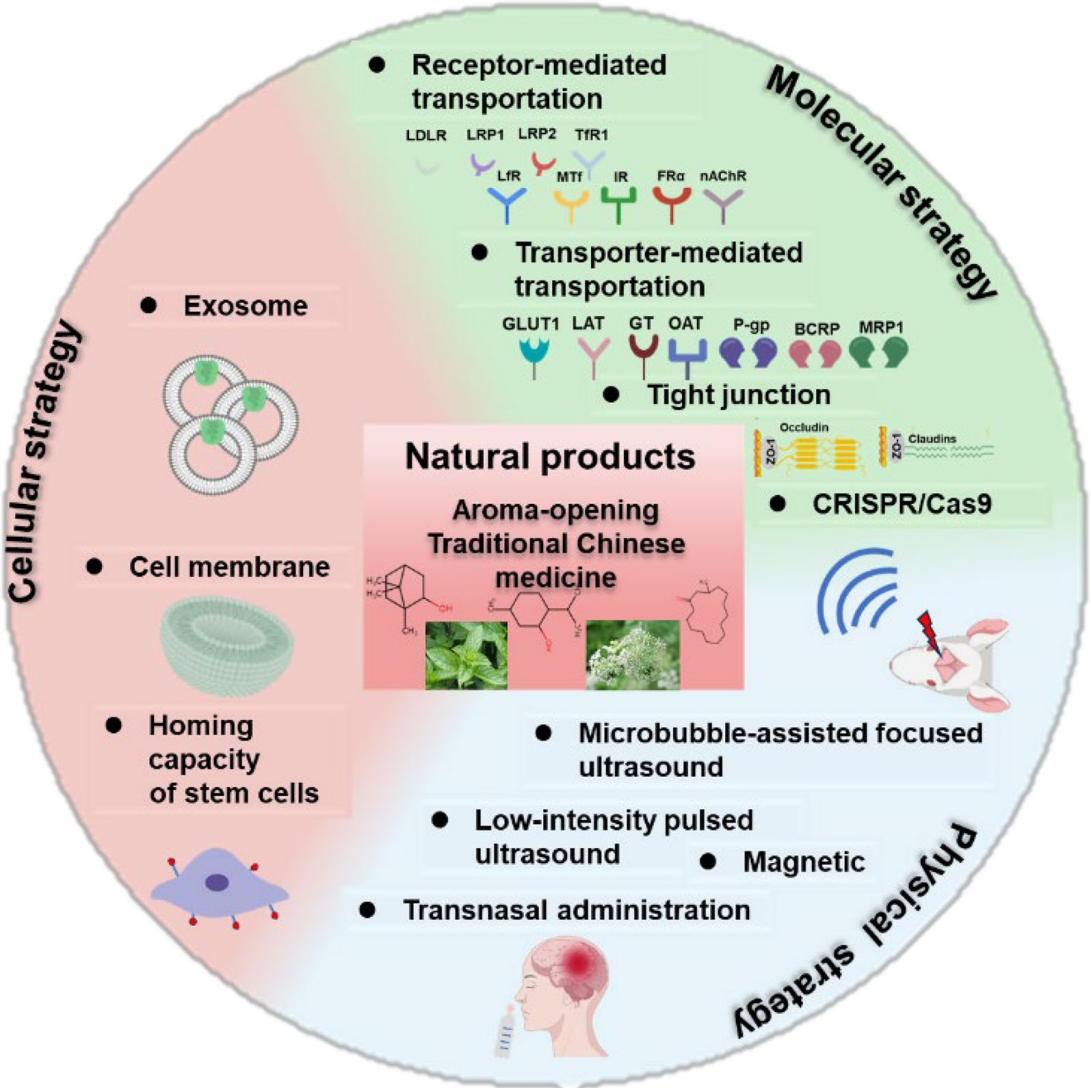

## Introduction

Neurological disorders, including brain tumors, Alzheimer's disease (AD), and Parkinson's disease, continue to pose significant challenges in the medical field [1–3]. The blood–brain barrier (BBB) serves as a critical protective mechanism for the brain, shielding it from external threats. However, this protective function prevents the majority of small molecule drugs and macromolecules (such as peptides, proteins, and gene-based drugs) entering the brain from the bloodstream, severely limiting the treatment of central nervous system (CNS) disorders [4]. Despite significant advances in drug target delivery, the effective delivery of therapeutics to the brain remains a major challenge in treating CNS disorders. Thus, developing targeted drug delivery systems capable of efficiently transporting therapeutics across the BBB is critical for treating CNS disorders.

The BBB is a highly selective permeable barrier, maintained through a complex interplay of proteins and cellular structures. Tight junction proteins form tight junctions (TJs) between endothelial cells in brain microvessels, restricting the non-selective passage of molecules [5]. Additionally, multiple receptor proteins, transporter proteins, and ion channels expressed on endothelial cells play crucial roles in regulating the transcellular transport of molecules and ions [6, 7]. Astrocytes and pericytes further contribute to the integrity of the BBB, with pericytes regulating barrier permeability through the secretion of extracellular matrix proteins, such as vitronectin, which interact with integrin receptors on endothelial cells [8]. Moreover, drug efflux transporter proteins, such as ABCB1 (P-glycoprotein, P-gp) and ABCG2 (Breast Cancer Resistance Protein, BCRP), expressed on the BBB, further limit drug delivery to the brain [9]. These physiological features highlight the potential for nanosystems to exploit receptor-mediated transport, transporter-mediated transport, and the regulation of tight junction-mediated transport for enhanced drug delivery.

The rapid development of nanosystems offers promising potential for treating CNS disorders. Due to their unique size effect (enhanced permeation and retention effect) and surface modification capabilities, nanosystems can more effectively penetrate the BBB, providing new hope for the treatment of brain diseases [10–12]. In the context of brain tumors, nanosystems enable targeted drug delivery, minimizing damage to healthy brain tissue while enhancing therapeutic efficacy. For neurodegenerative diseases such as AD, nanosystems can transport therapeutic drugs or gene-editing tools directly to affected brain cells, facilitating the repair of damaged neurons or reducing the accumulation of abnormal proteins [13, 14]. Additionally, nanosystems can be utilized for the early detection of neurological disorders by delivering targeted contrast agents or biomarkers, thereby improving diagnostic accuracy and sensitivity [15, 16]. However, traditional drug nanosystems often face challenges such as potential toxicity, limited biocompatibility, and lack of multifunctionality, whereas natural products offer advantages like inherent biocompatibility and multi-targeting, making them a promising alternative for effective and safer CNS drug delivery.

Recent advancements have highlighted the potential of natural products in the therapeutic management of CNS [17, 18]. Certain natural small molecules, such as volatile components, omega-3 polyunsaturated fatty acids, polyphenols, and terpenoids, have been reported to cross the BBB [19–21]. The mechanisms underlying this ability may involve interactions with receptor proteins, suppression of efflux protein activity, and regulation of tight junction protein expression [22]. These mechanisms underscore the significant role of natural products in the treatment of brain disorders. Importantly, the combination of nanosystems with natural products represents an emerging area of research. The combination of aromatic Chinese medicines with modern drug nanosystems, such as nanocarriers, has shown significant potential. For example, drug-carrying liposomes modified with borneol and menthol have improved drug distribution in the brain [23, 24]. This integration not only enhances the efficacy of traditional medicines but also provides new insights into the design of brain-targeted nanosystems.

**Fig. 1:**
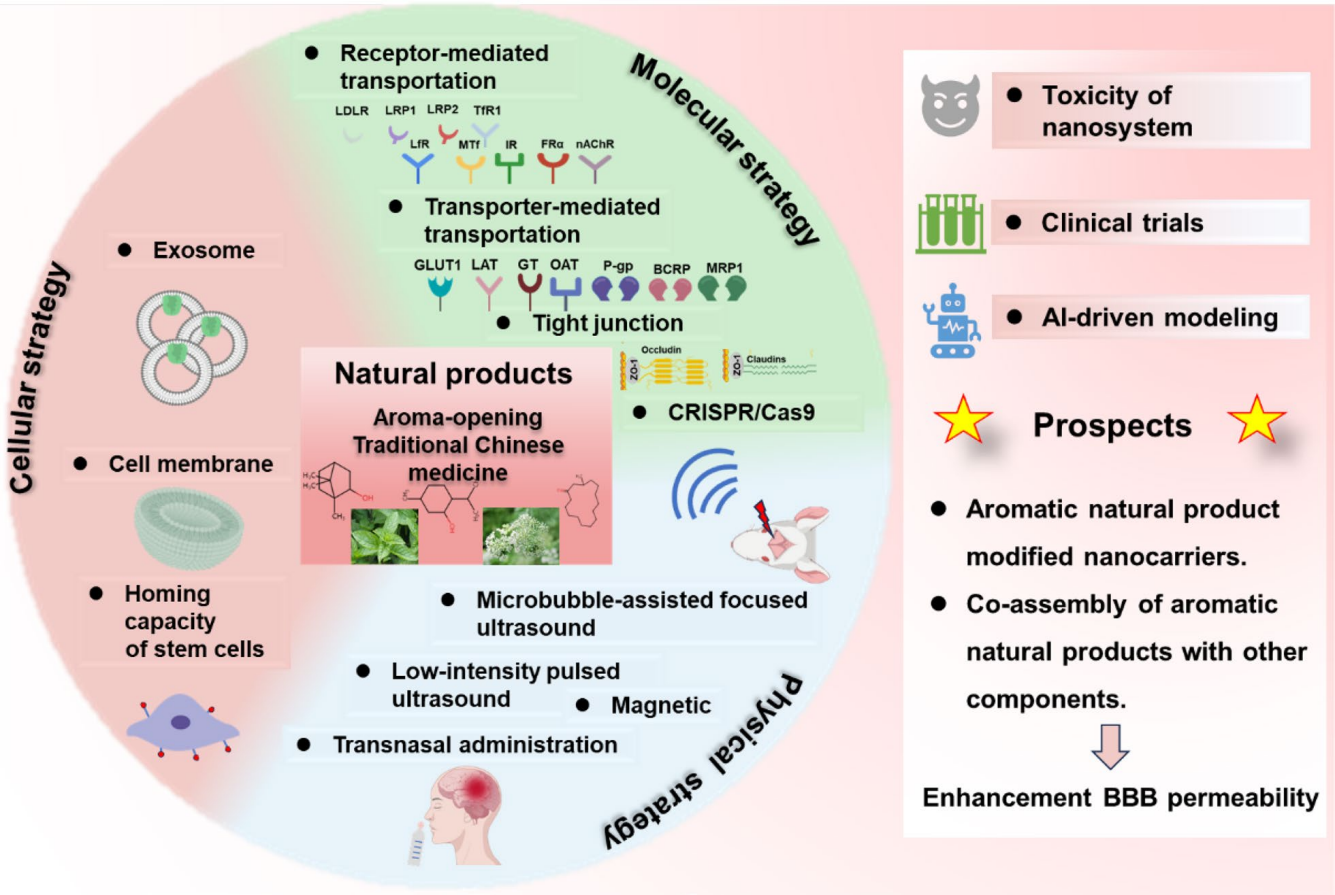
Multiple strategies-mediated BBB transport and natural products-regulated BBB permeability research prospects

This review systematically examines contemporary brain-targeted drug delivery strategies through three primary modalities: (1) molecular approaches including receptor-mediated transport, transporter-mediated uptake, tight junction modulation, adsorptive-mediated transcytosis, and CRISPR-enabled BBB penetration; (2) cellular strategies utilizing exosomes, cell membrane coatings, and stem cell homing mechanisms; and (3) physical methods incorporating focused ultrasound with microbubbles, low-intensity pulsed ultrasound, and magnetic field-guided delivery, along with alternative intranasal administration routes. We comprehensively analyze their therapeutic applications in CNS disorders (Table 1), comparatively evaluate their advantages and limitations (Table 2), and specifically explore aromatic Chinese herbs' BBB-modulating effects via tight junction protein and transporter regulation. Our discussion further evaluates nanosystem toxicity, clinical translation progress, and AI-driven BBB permeability prediction, concluding with challenges and future directions for natural product-derived nanocarriers in CNS-targeted delivery (Fig. 1).

**Table 1:** Blood–brain barrier targeted delivery strategies

No	BBB-targeting strategies	Mechanism of action	Delivery system	Therapeutic drug	Disease/organ/model	Reference
1	Receptor-mediated transportation	TfR	Transferrin-drug conjugate	L-arginine-coated iron oxide	Alzheimer's disease	[35]
2		TfR	Transferrin modified liposomes	Temozolomide	Glioblastoma	[58]
3		TfR	Transferrin modified liposomes	Adriamycin	Glioblastoma	[169]
4		TfR	Transferrin modified liposomes	Cisplatin	Glioblastoma	[39]
5		TfR	Transferrin modified liposomes	Osthole	Alzheimer's disease	[42]
6		TfR	Transferrin modified liposomes	Caffeic acid	Alzheimer's disease	[43]
7		TfR, nAChRs	Transferrin and rabies virus glycoprotein peptide modified liposomes	Therapeutic nucleic acid	Brain	[170]
8		TfR, PFVYLI or R9F2	Transferrin and cell-penetrating peptide modified liposomes	plasmid DNA	Brain	[171]
9		TfR	Transferrin modified liposomes	Rutin	Alzheimer's disease	[44]
10		TfR	CpG oligonucleotides anchored endogenous serum exosomes	Tanshinone IIA and glycyrrhizic acid	Glioblastoma	[108]
11		IGF1R	IGF1R-neurotensin, galanin conjugate	Neurotensin, galanin	Brain	[65]
12		IR	83–14 monoclonal antibody-modified solid lipid nanoparticles	Saquinavir	Brain	[66]
13		FRα	Folate-coupled exosomes	Temozolomide	Glioblastoma	[68]
14		FRα	Folic acid-modified hollow titanium dioxide nanospheres	Temozolomide	Glioblastoma	[69]
15		FRα	FRα modified polyethylene glycol-polycaprolactone	Folic acid	Brain	[70]
16		LfR	Lactoferrin-conjugated linoleic acid conjugate	Linoleic acid	Alzheimer's disease	[55]
17		LfR	Lactoferrin modified hollow mesoporous copper sulfide nanoparticles	Temozolomide	Glioblastoma	[172]
18		LfR	Lactoferrin conjugated heparin	Heparin	Glioblastoma	[56]
19		LfR	Lactoferrin conjugated ultra-small size with large pore silica nanoparticles	Doxorubicin	Glioblastoma	[57]
20		LfR	Lactoferrin modified ultra-small large pore silica nanoparticles	Temozolomide	Glioblastoma	[58]
21		LfR	Lactoferrin conjugated resveratrol-loaded PLGA nanoparticles	Resveratrol	Parkinson's disease	[59]
22		LfR	PEGylated-lactoferrin modified Chlorin e6 (Ce6) and glutathione coated-AuNPs	Ce6, AuNPs	Glioblastoma	[173]
23		LfR	Rabies virus glycoprotein and lactoferrin -grafted liposomes	IAP antagonists, AZD5582 and SM-164	Glioblastoma	[174]
24		LfR	Lactoferrin modified Au-Bi_2_Se_3_ nanodot	Au-Bi_2_Se_3_	Parkinson's disease	[175]
25		LfR	Lactoferrin modified YOF: Nd^3+^ as core, MnO_2_ as shell, and further loading photosensitizer and glucose oxidase	Indocyanine green and glucose oxidase	Glioblastoma	[176]
26		LfR	Lactoferrin-conjugated micelles	Glutaminyl cyclase inhibitor 8	Alzheimer's disease	[177]
27		LfR	Lactoferrin and muscone dual-modified liposomes	Docetaxel	Glioblastoma	[62]
28		LfR	Lactoferrin modified riluzole-loaded nanostructured lipid carriers	Riluzole	Amyotrophic lateral sclerosis	[178]
29		LfR	Black phosphorus nanosheets containing the lactoferrin and loaded with paeoniflorin	Paeoniflorin	Parkinson's disease	[60]
30		LfR	Lactoferrin modified puerarin-loaded graphene oxide	Puerarin	Parkinson's disease	[61]
31		LfR	Lactoferrin modified Au complex (C2)	Au complex (C2)	Glioblastoma	[179]
32		LfR	Lactoferrin/CD133 antibody conjugated nanostructured lipid carriers	Temozolomide	Glioblastoma	[180]
33		LRP1	Lactoferrin modified	Dihydroartemisinin and the indocyanine green	Glioblastoma	[181]
34		LDLR	ApoE-modified nano-micelles	Oridonin and phillyrin	Alzheimer's disease	[52]
35		LRP1	ROS-responsive biomimetic exosome-liposome hybrid nanovesicles	β-site amyloid precursor protein cleaving enzyme-1 and TREM2 plasmid gene	Alzheimer's disease	[112]
36		MTf	MA crosslinked etoposide‐loaded solid lipid nanoparticles	Etoposide	Glioblastoma	[182]
37	Transporter-mediated transportation	GLUT1	Glycosylated "triple-interaction" stabilized polymeric siRNA nanomedicine	Polymeric siRNA	Alzheimer's disease	[76]
38		GLUT1	Mannose modified PLGA-PEG skeleton	Fingolimod	Alzheimer's disease	[77]
39		LAT1	LAT1-connected nipecotic acid prodrug	Nipecotic acid	Epilepsy	[79]
40		LAT1	Valine conjugated chitosan modified PCL-PEG-PCL triblock copolymers	Rivastigmine and quercetin	Alzheimer's disease	[80]
41		Glutathione transporter	Glutathione-conjugated magnetic nanoparticles	Paclitaxel	Glioblastoma	[82]
42		Glutathione transporter	Glutathione pegylated liposomes	Methylprednisolone	Neuroinflammation	[83]
43		nAChR	RVG29 peptide and PEG-modified nanocarrier	Therapeutic gene and peptide	Alzheimer's disease	[183]
44	Regulate tight junction	ZO-1, integrin and selectin	Metastatic melanoma cell membrane	siRNA complexed polyethyleneimine xanthate	Glioblastoma	[184]
45		ZO-1、claudin-5、occludin	GBM-cell membrane camouflaged	Temozolomide and cisplatin	Glioblastoma	[100]
46	Cellular strategy	Endocytosis	Exosomes produced by M2-type macrophages	DNase 1	Ischaemic stroke	[185]
47		Tumor tendency of MSCs	Silica nanorattle-doxorubicin-anchored mesenchymal stem cells	Doxorubicin	Glioblastoma	[186]
48		Erythrocyte membrane	Erythrocyte membrane-modified core–shell upconversion nanoparticle	Curcumin	Alzheimer's disease	[114]
49		Macrophage plasma membrane	Macrophage plasma membrane decorated DSPE-PEG loaded IR-792 nanoparticles	IR-792	Glioblastoma	[187]
50	Physical strategy	Focused ultrasound/microbubble	mRNA encapsulated-lipid nanoparticles	mRNA	Brain	[188]
51		Focused ultrasound/microbubble	Superparamagnetic iron oxide and doxorubicin to prepare microbubbles	Doxorubicin	Glioblastoma	[189]
52		Focused Ultrasound	——	Etoposide	Glioblastoma	[190]
53		Focused ultrasound	——	Panobinostat	Diffuse midline glioma mouse model	[191]
54	Intranasal delivery	Intranasal administration	Black phosphorus loaded with methylene blue is incorporated into thehydrogel	Methylene blue	Alzheimer's disease	[192]
55		Intranasal administration	Edaravone-loaded poly (lactic-co-glycolic acid)-based polymeric nanoparticles	Edaravone	Amyotrophic lateral sclerosis	[129]
56		Intranasal administration	Aleuria aurantia lectin and β-amyloid -binding peptides modified PEGylated dendrigraft poly-l-lysines	Small interfering RNA of β-site precursor protein cleaving enzyme-1 and rapamycin	Alzheimer's disease	[193]
57		Intranasal administration	mPEG-PCL encapsulate curcumin	Curcumin	Intracerebral hemorrhage	[194]
58		Intranasal administration	Dolutegravir-loaded nanoemulsion-based in situ gel	Dolutegravir	Neuro AIDS	[195]
59	Aroma-opening natural products	P-gp and tight junction proteins	Borneol-modified schisandrin B	Schisandrin B	Alzheimer's disease	[136]
60			Borneol-modified docetaxel plus tetrandrine micelles	Docetaxel, tetrandrine	Glioblastoma	[137]
61			Menthol-modified quercetin liposomes	Quercetin	Alzheimer's disease	[24]
62			Menthol-modified BSA nanoparticles	Albendazole-loaded menthol-modified BSA-silver	Glioblastoma	[139]
63			Muscone/RI7217 co-modified upward messenger DTX liposomes	Docetaxe	Glioblastoma	[142]
64			β-asarone and levodopa co-administration	Levodopa	Parkinson's disease	[145]

**Table 2:** The advantages and disadvantages of different brain-targeting methods

Strategy	Brain-targeting methods	Advantages	Disadvantages
Molecular strategy	Receptor-mediated transportation	High specificity (such as TfR, LDLR targeting)	Poor human translation
	Transporter-mediated transportation	Utilizes endogenous nutrient pathways; Bypasses efflux pumps	Nutrient competition effects; Disease-dependent expression changes
	Tight junction protein	Reversible BBB opening; Enhances paracellular transport	Risk of neuroinflammation
	Adsorption endocytosis	Strong electrostatic attraction	Lysosomal degradation
	CRISPR-based BBB modulation	Long-term BBB permeability enhancement; Potential for precise gene regulation (such as tight junction disruption)	Off-target editing risks; Immune response; Ethical/safety concerns
Cellular strategy	Exosome-based	Native BBB crossing ability; Low immunogenicity	Production scalability issues; Drug loading efficiency < 5% typically; Poor targeting control
	Cell membrane coating	Retains source cell tropism; Evades immune clearance	Limited penetration beyond vasculature
	Stem cells homing	Pathotropism to lesions	Risk of tumorigenesis; Ethical constraints
Physical strategy	Focused ultrasound with microbubbles	Spatial precision (mm-scale); Immediate effect	Requires specialized equipment; Microhemorrhage risk;
	Magnetic field-guided delivery	Deep tissue penetration; Real-time tracking possible	Requires superparamagnetic materials; Gradient field limitations; Potential tissue heating
	Intranasal administration	Bypasses BBB completely; Rapid CNS delivery	Olfactory toxicity
Aroma-opening natural products	Combined with other drugs; modified nanocarrier	Multi-target synergy; Reversible regulation; therapeutic effect	Dose-dependent biphasic effect; Standardization challenges

## Structure of the BBB and targeted delivery strategies

**Fig. 2:**
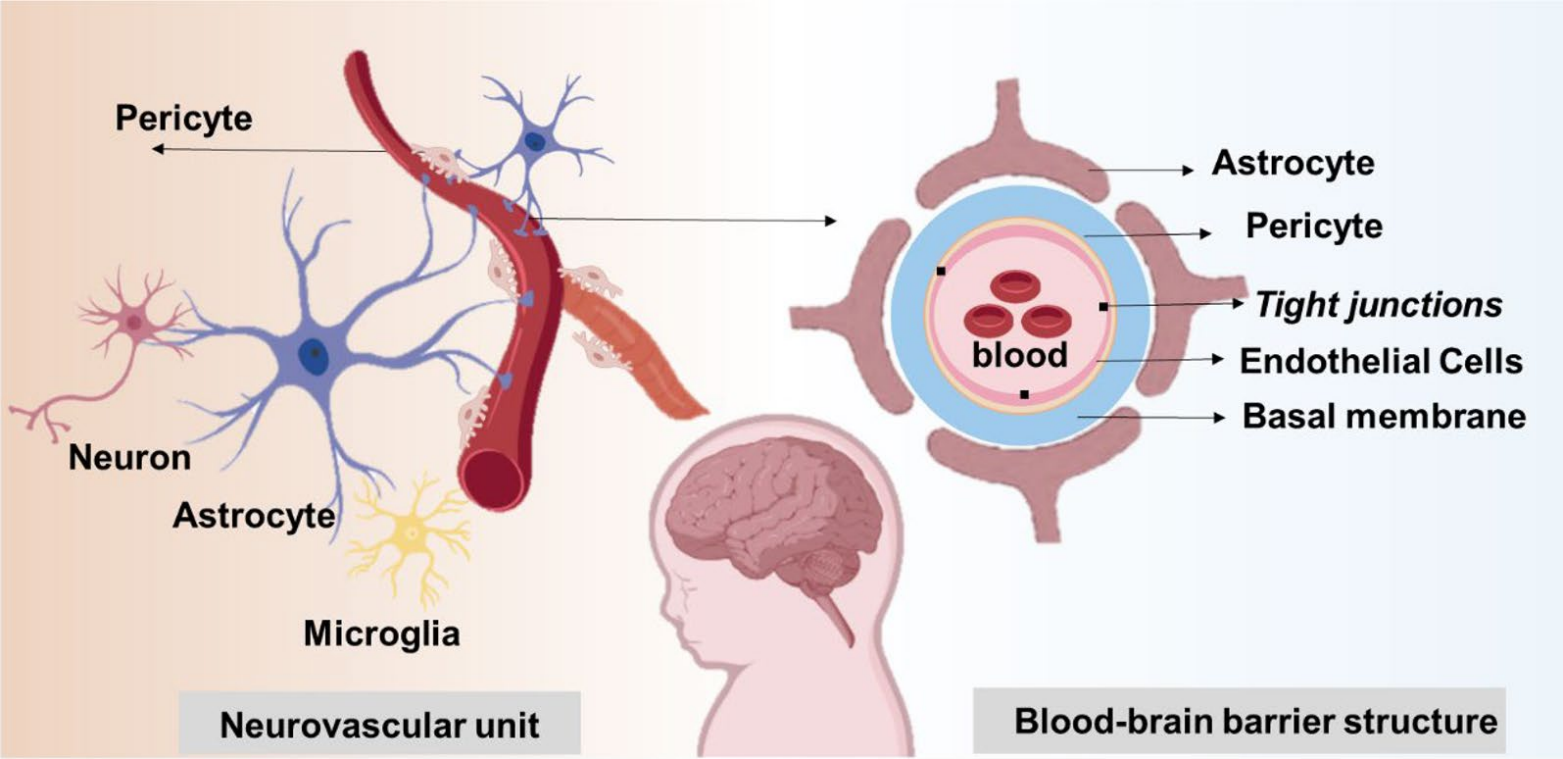
Neurovascular unit and BBB structure

The BBB is a specialized physiological barrier that separates blood from brain tissue. It is primarily composed of endothelial cells, astrocytes, pericytes, basement membranes, and junctional complexes, including TJs and adhesion junctions (AJs) [25] **(**Fig. 2**)**. Brain microvascular endothelial cells (BMECs) constitute a central component of the BBB. These cells are interconnected by TJs, which are formed by transmembrane proteins (such as occludin, Claudin-3, Claudin-5) [26] and peripheral proteins (such as Zona Occludens-1, 2, 3; ZO-1, ZO-2, ZO-3) [27]. The presence of TJs confers the BBB with high selectivity, effectively preventing the passage of most water-soluble substances and macromolecules through the paracellular pathway [28]. BMECs also have some special properties such as lack of windowing, low levels of non-specific cytosis (cytosolic drinking) and low paracellular diffusion capacity [29]. Furthermore, BMECs express a variety of membrane receptors and transporters that facilitate the active transport of essential nutrients and metabolites across the BBB [30]. The basement membrane, secreted by both BMECs and astrocytes, is another critical component of the BBB. It is primarily composed of collagen IV, laminin-1, 2, 4, and 5, as well as glycoproteins [31]. This structure plays a vital role in maintaining the structural integrity of the BBB. Astrocytes extend their end feet into the perivascular space, forming close associations with BMECs. They modulate the permeability and transport functions of endothelial cells through the secretion of various signaling molecules [32]. Pericytes, which overlay the basement membrane of BMECs, are also integral to the BBB. It forms the neurovascular unit together with cerebral microvascular endothelial cells and astrocytes, and participates in the formation and regulation of the BBB [33]. Additionally, the results showed that matrix metalloproteinases (MMPs) along with their inhibitors (Tissue inhibitor of metalloproteinase-1, TIMP-1) play significant roles in the regulation of the BBB [34].

### Molecular strategies

**Fig. 3:**
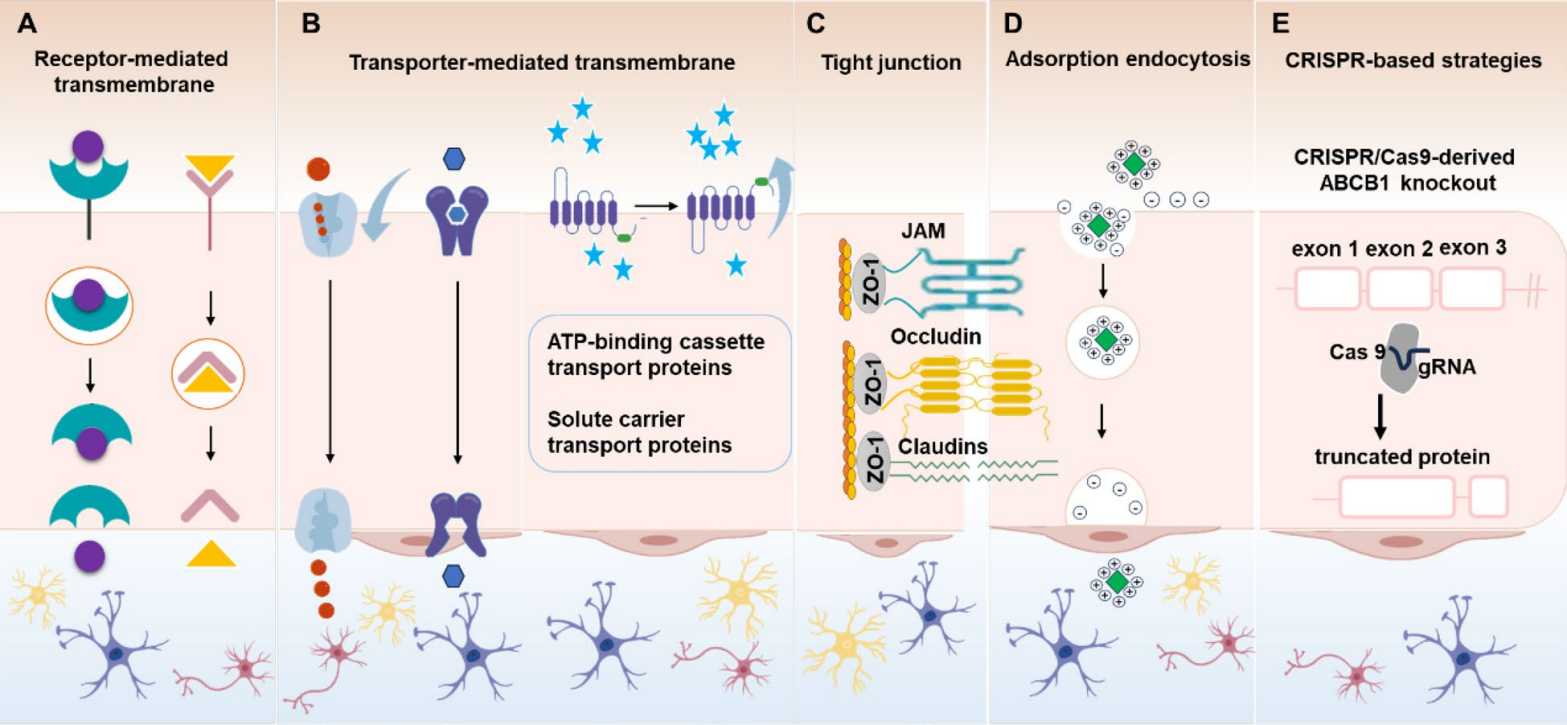
Molecular strategies mediated delivery of targeted BBB, including (**A**) receptor-mediated transmembrane; **B** transporter-mediated transmembrane; **C** regulate tight junction; **D** adsorption endocytosis-mediated transmembrane; **E** CRISPR-based strategies for BBB modulation (using ABCB1 knockout as an example)

The surface receptors, transporters, tight junction proteins and adsorption endocytosis associated with the BBB are essential for preserving its integrity and modulating the transport of substances into and out of the CNS. The targeted BBB delivery approaches mediated by these molecular strategies are systematically illustrated in Fig. 3. In the subsequent sections, we will provide a detailed examination of the receptors, transporters (influx transporters and efflux transporters), tight junction proteins, adsorption endocytosis and CRISPR-based strategies. Additionally, the advantages and disadvantages of these strategies are also assessed.

#### Receptor-mediated transportation through BBB

Some specific receptors highly expressed on the BBB provide an important breakthrough for brain-targeted delivery strategies. By exploiting the mediating effects of these receptors, nanocarriers can facilitate trans-BBB drug delivery, thereby providing novel therapeutic approaches for CNS diseases. In this review, we systematically summarize the receptors highly expressed on the BBB and their associated trans-BBB delivery strategies. These receptors include the transferrin receptor, low-density lipoprotein receptor, lactoferrin receptor, melanotransferrin, insulin receptor, folate receptor, and N-acetylcholine receptor, among others.

##### Transferrin receptor

**Fig. 4:**
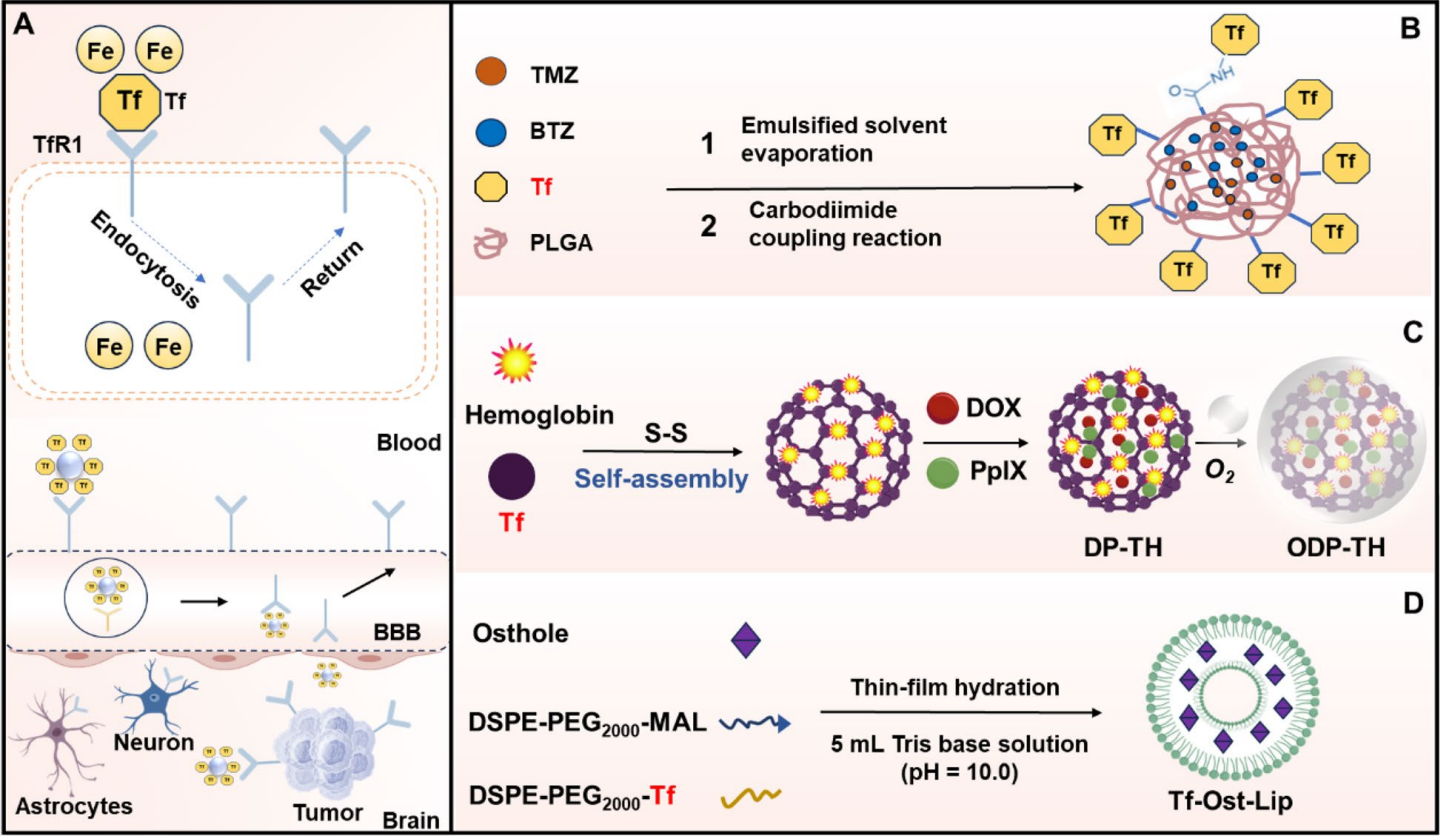
Transferrin receptor structure and transferrin receptor-mediated strategies cross the BBB. **A** Transferrin receptor-mediated cross the BBB. **B** Transferrin-coupled TMZ + BTZ-loaded PLGA nanoparticles. **C** Transferrin-coupled hemoglobin carriers for targeted delivery of PpIX and DOX across the BBB. **D** Transferrin-modified osthole liposomes

The expression level of transferrin receptor 1 (TfR1) is significantly elevated in both the BBB and tumor cells compared to normal tissues. Consequently, TfR1 has emerged as a critical target for tumor-specific therapies and interventions targeting neurological disorders. Drug delivery systems designed for TfR1-targeted therapy primarily encompass transferrin (Tf), anti-TfR1 antibodies, TfR1-binding peptides and various biomolecules with specific affinity for TfR1. Upon binding, these agents facilitate cellular uptake or enable traverse the BBB via receptor-mediated transcytosis (Fig. 4A). Notably, substantial advancements have been achieved in leveraging TfR for targeted drug delivery to the brain.

Drug-transferrin conjugates represent a class of compounds in which pharmacological agents are covalently attached to Tf, leveraging its inherent biological properties to achieve targeted drug delivery to specific tissues or cell populations. Conjugating small molecule drugs with Tf enhances the permeability of hydrophilic chemotherapeutic or neurotherapeutic agents into tumor cells or across the BBB. Choi et al*.* designed the Tf -conjugated melittin-loaded L-arginine-coated iron oxide nanoparticles (Tf-MeLioNs), a nanomedicine with a "core–shell" structure, for the treatment of AD. The synthesis involved co-precipitating iron salts with L-arginine to form iron oxide nanoparticles (IONPs), followed by surface modification with L-arginine (LioNs). Melittin was then bound to the surface of LioNs via electrostatic interactions or covalent bonds. Finally, Tf was conjugated to melittin using a carbodiimide coupling agent, resulting in Tf-MeLioNs. The nanomedicine effectively delivered melittin to brain lesions, ameliorating pathological changes in AD model mice by reducing amyloid plaque formation, inhibiting microglial activation, and regulating Aβ metabolism-related proteins. These findings suggested that Tf-MeLioNs hold promise for AD treatment [35]. In another study, temozolomide (TMZ) and bortezomib (BTZ)-loaded poly(lactic-co-glycolic acid) (PLGA) nanoparticles were prepared using a single emulsion-solvent evaporation method. Tf was subsequently conjugated to the nanoparticle surface via a carbodiimide coupling reaction. Efficacy and safety evaluations demonstrated that this nanomedicine could overcome glioma drug resistance and reduce side effects [36] (Fig. 4B). Additionally, researchers developed a protein hybridization platform (ODP-TH) utilizing Tf as a multi-class solid tumor identifier, in conjunction with hemoglobin (Hb) to facilitate oxygen delivery. Subsequently, the photosensitizer protoporphyrin IX (PpIX) and the chemotherapeutic agent doxorubicin (DOX) were encapsulated and linked via glutathione-responsive disulfide bonds. This approach, combining homologous targeting with oxygen supplementation, enabled a synergistic photodynamic-chemotherapeutic strategy for treating malignant tumors, effectively addressing hypoxia and chemotherapy resistance [37] (Fig. 4C).

Transferrin-modified liposomes (Tf-LPs) represent a novel drug delivery system. Numerous studies have reported the use of Tf-LPs loaded with various drugs, including chemotherapeutic agents and neuroprotective agents, for treating brain tumors and neurodegenerative diseases. For example, Tf-LPs loaded with TMZ [36], DOX [38], and cisplatin (CDDP) [39] demonstrated enhanced uptake in brain tumor cells and improved tumor suppression in animal models compared to conventional administration. Similarly, Tf-LPs loaded with neuroprotective agents such as edaravone, minocycline, and doxycycline have shown promise in neurodegenerative disease research [40, 41].

Moreover, certain natural small molecule compounds loaded into Tf-LPs exhibit excellent BBB permeability and therapeutic potential for brain diseases. For example, Kong et al. constructed a transferrin-modified osthole liposome (Tf-Ost-Lip), which improved the bioavailability of imperatorin and enhanced BBB penetration. This formulation exerted neuroprotective effects and ameliorated AD-related pathology and cognitive function in AD mice [42] (Fig. 4D). Other studies have demonstrated that Tf-LPs loaded with caffeic acid [43], rutin [44] and vincristine [45] enhanced the BBB permeability of natural products. In a recent investigation, biomimetic blood exosomes and tLyp-1-modified liposomes were engineered to incorporate penetrated hybrid nanovesicles co-loaded with salvianolic acid B and cryptotanshinone. These vesicles significantly improved BBB traversal and CNS access through targeted interaction with the TfR. Glioma cell endocytosis, guided by the tLyp-1 peptide, induced cytotoxic responses and exhibited anti-angiogenic properties, highlighting their potential for cancer therapy [46].

In conclusion, Tf-modified drug delivery systems present considerable advantages in the management of CNS disorders. These advantages include: (1) Enhanced targeting: TfR1 is abundantly expressed in BMECs and tumor cells, making TfR1-based drug delivery systems highly specific. (2) Diverse options for drug carriers: TfR1-targeted therapies utilize a range of carriers, including Tf, anti-TfR1 antibodies, and TfR1-binding peptides. This diversity provides flexibility in selecting appropriate carriers for different pharmacological agents. However, as a protein, Tf has the potential to induce immune responses. In some cases, Tf-LPs may be recognized as foreign entities by the immune system, triggering immune reactions that could compromise the safety and efficacy of these formulations.

##### Low density lipoprotein receptor (LDLR)

**Fig. 5:**
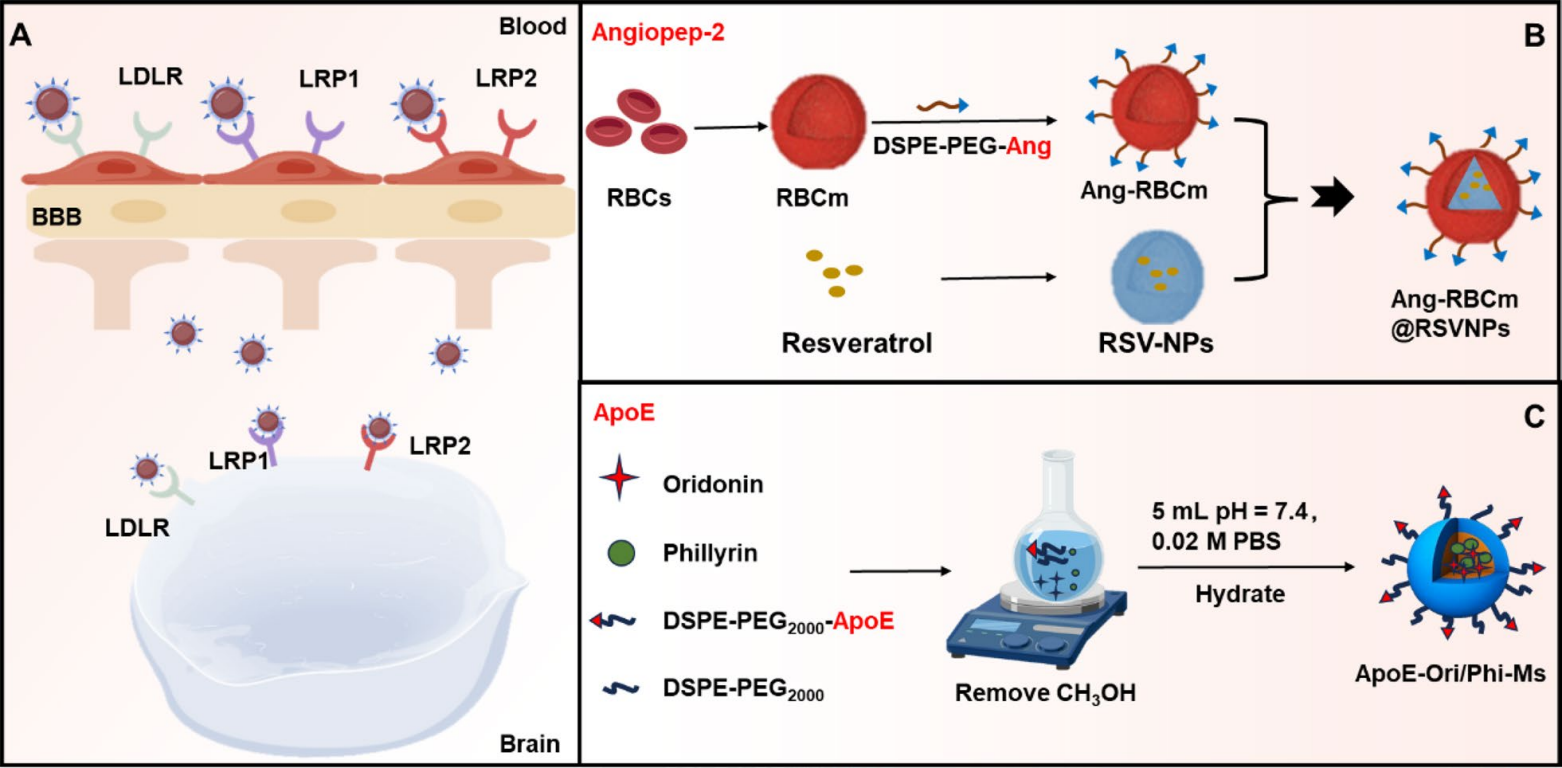
Low-density lipoprotein receptor structure and low-density lipoprotein receptor-mediated strategies cross the BBB. **A** Multiple low-density lipoprotein receptors expressed at the BBB. **B** Ang-2-modified red blood cell membrane (Ang-RBCm)-encapsulated resveratrol (RSV) nanoparticles. **C** ApoE-modified oridonin (Ori) and phillyrin (Phi) nanomicelles

The low-density lipoprotein receptor (LDLR) is a transmembrane protein composed of 839 amino acids, primarily responsible for modulating the endocytosis of LDL and is highly expressed at the BBB. Its specific ligands include LDL and apolipoprotein (Apo). Commonly used functional groups for targeting LDLR include Angiopep-2 (Ang-2) and apolipoprotein E (ApoE), which provide efficient pathways for nanomedicine delivery across the BBB. Low-density lipoprotein receptor-related protein 1 (LRP1), a multifunctional transmembrane protein, plays a critical role in the BBB. Numerous investigations have elucidated the distinctive expression profile of LRP1 within the BBB and its essential physiological roles. The BBB expresses a variety of LDL receptors, as illustrated in Fig. 5A.

LDL-modified nanomedicines demonstrate remarkable specificity in targeting CNS disorders. For instance, one study explored a drug delivery system for gliomas by encapsulating vincristine sulfate in low-density lipoprotein particles (T7-LDL) modified with T7 peptide. LDL acts as an endogenous lipid transport carrier that binds specifically to the LDLR on brain endothelial cells and glioma cells. T7 peptide is a TfR ligand capable of crossing the BBB and targeting gliomas. The dual-targeting approach of T7 peptide and LDL significantly enhanced glioma targeting efficiency. T7-LDL loaded with vincristine demonstrated optimal anti-glioma effects both in vitro and in vivo, highlighting its potential as a drug delivery system for glioma therapy [47]. In conclusion, drugs encapsulated in LDL-modified nanoparticles could be delivered across the BBB through LDL receptor-mediated cytosis, which has been employed in therapeutic research for various brain diseases [48, 49]. Ang-2, a peptide targeting low-density lipoprotein receptor-related protein 1 (LRP-1), has been utilized in the design of Ang-2-modified red blood cell membrane (Ang-RBCm)-encapsulated resveratrol (RSV) nanoparticles (Ang-RBCm@RSV NPs). These nanoparticles effectively penetrated the BBB and accumulated in the brain, exhibiting enhanced anti-addiction and neuroprotective effects [50] (Fig. 5B). ApoE, primarily produced by astrocytes, acts as a ligand interacting with various receptors involved in lipoprotein transport across the BBB. Researchers have designed acid-sensitive bionic nanocarriers based on ApoE peptide-modified erythrocyte membranes for the delivery of Bcl-2/Bcl-xl and Mcl-1 inhibitors to treat glioblastoma (GBM). These nanocarriers effectively crossed the BBB, targeted brain gliomas, inhibited tumor growth and prolonged the survival cycle of hormonal mice [51]. In addition, ApoE-modified oridonin and phillyrin nanomicelles (ApoE-Ori/Phi-Ms) traversed the BBB under the guidance of the brain-targeting peptide ApoE (Fig. 5C). This approach increased the effective drug concentrations in the brain, improved cognitive performance, reduced Aβ deposition, attenuated neuroinflammation and oxidative stress, inhibited aberrant activation of astrocytes and microglia, and rescued neuronal apoptosis in AD mouse models [52]. To facilitate the delivery of neuroprotectants, Wang et al. developed a biomimetic nanomotor capable of traversing the BBB and penetrating deeper ischemic and hypoxic brain regions. It was achieved through the active targeting properties of apo-lactoferrin (Apo-LF) and self-propelling motility, demonstrating significant neuroprotective effects in deeper brain regions [53]. Despite the potential of LRP receptor-mediated transport for BBB traversal, nanocarrier-based delivery systems still face challenges in achieving efficient penetration. For instance, carrier modification and design must be precisely controlled to ensure efficient receptor binding and internalization while avoiding immune system recognition and clearance.

##### Lactoferrin receptor

Lactoferrin Receptor (LfR) is highly expressed in brain endothelial cells, capillaries and neurons associated with neurodegenerative diseases, which has been applied for brain-targeted drug delivery via BBB receptor-mediated cytosolic targeting [54].

**Fig. 6:**
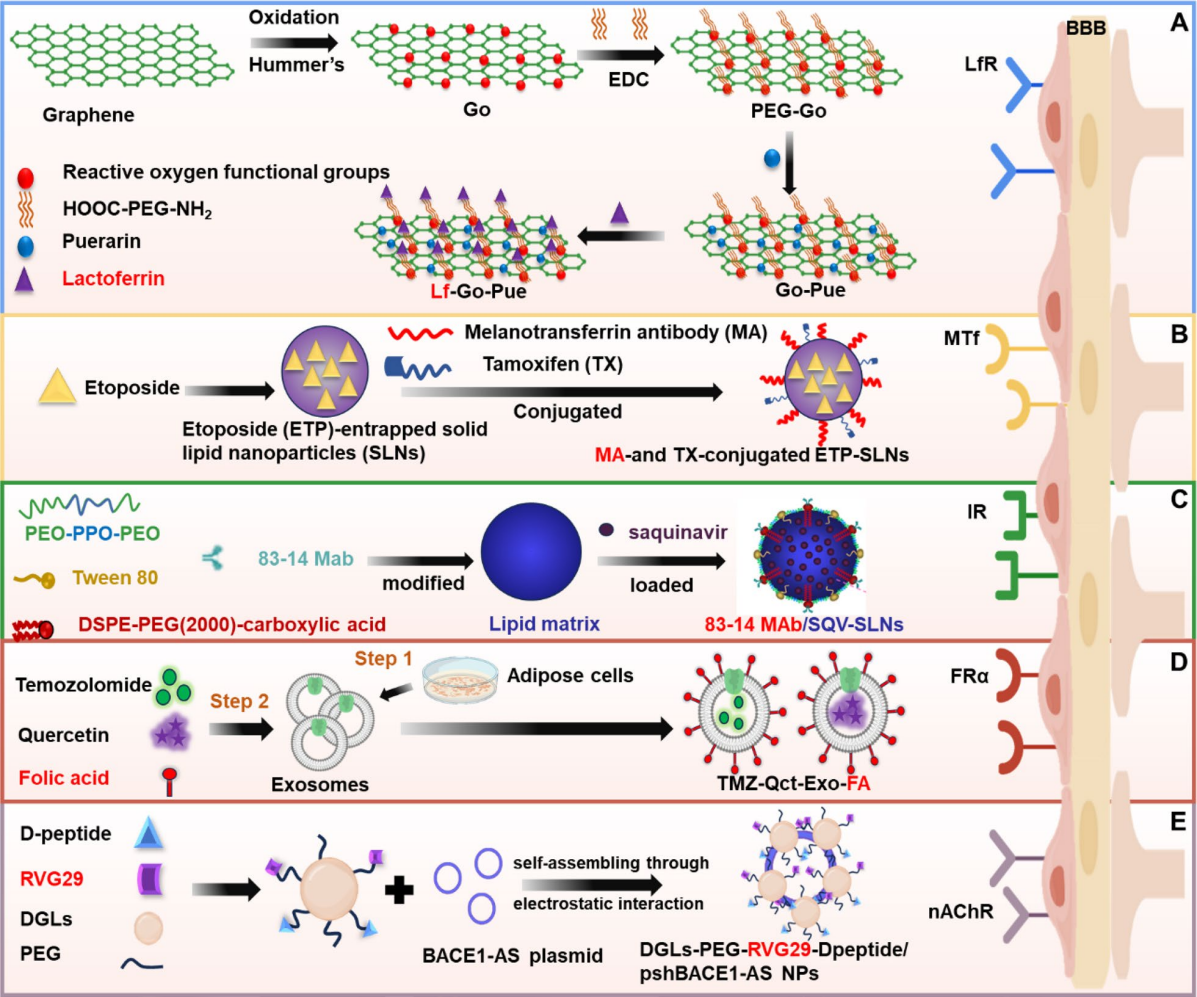
LfR, MTf, IR, FRα and nAChR receptor-mediated strategies for crossing the BBB. **A** LF-modified graphene oxide (GO) nanosheets loaded with puerarin (Pue) through the BBB via LfR. **B** Melanotransferrin antibody (MA) and tamoxifen (TX)-conjugated solid lipid nanoparticles (SLNs) encapsulated with etoposide (ETP) cross the BBB via MTf. **C** 83–14 monoclonal antibody (MAb)-modified solid lipid nanoparticles (SLNs) loaded with saquinavir (SQV) to improve the brain-targeting delivery via IR. **D** Folic acid (FA)-conjugated exosomes encapsulated with TMZ and quercetin (Qct) cross the BBB via FRα. **E** RVG29 and D-peptide-modified DGLs complexed with plasmid DNA encoding BACE1-AS shRNA yielding DGLs-PEG-RVG29-Dpeptide/pshBACE1-AS NPs

Lactoferrin (LF)-conjugated linoleic acid (CLA) micelles, fabricated via carbodiimide coupling and loaded with CLA, demonstrated enhanced in vivo biorelease in brain tissue while improving cognitive function in an aluminum chloride-induced AD animal model [55]. LF forms amide bonds with heparin, enabling its absorption by the small intestine after oral administration and subsequent delivery to brain tumors via LfR-mediated transport [56]. Additionally, LF-modified nanocarriers have effectively solved the BBB permeability challenges of DOX [57] and TMZ [58]. These nanocarriers demonstrated enhanced cellular internalization and significant tumor growth inhibition in vivo. Similarly, the properties of LF targeting the BBB and tumors were utilized to deliver natural products also improved their bioavailability in the brain. LF-modified nanocarrier carriers loaded with RSV, puerarin (Fig. 6A) and paeoniflorin greatly enhanced the neuroprotective effects of these components in a Parkinson's disease model and increased their brain bioavailability [59–61]. Notably one study reported that muscone-modified liposomes facilitated BBB traversal. Dual modification of liposomes with LF and muscone, loaded with docetaxel (DTX), enhanced brain delivery, providing a novel approach for natural product-modified nanomedicine research [62].

##### Melanotransferrin

Melanotransferrin (MTf) is a surface protein found on melanoma cells. It has been employed as a drug delivery vehicle for GBM treatment. Melanotransferrin antibody (MA)-coupled solid lipid nanoparticles have been used to deliver the anticancer drug etoposide across the BBB (Fig. 6B). Immunochemical staining revealed that MA triggered melanotransferrin-mediated transcytosis and inhibited the growth of U87MG cells [63].

##### Insulin receptor

The high expression of insulin receptor (IR) on brain microvascular endothelial cells provides a new pathway for brain drug delivery. Brain-targeted drug delivery can be achieved using insulin or insulin analogs as ligands, which bind to drugs to form conjugates that traverse the BBB via IR-mediated transport. Researchers have genetically engineered insulin fusion proteins capable of targeting hippocampal neurons. This innovative technique exploited the natural tendency of insulin to accumulate in hippocampal neuronal tissues, enabling targeted drug delivery and opening new avenues for treating neurodegenerative diseases such as AD [64]. Progress has also been made in monoclonal antibody research. For instance, neurotensin and galanin were coupled to the single-domain antibody IGF1R5 (sdAb IGF1R5), which targeted the insulin-like growth factor-1 receptor (IGF1R) to deliver these peptides to the brain, producing cooling and analgesic effects [65]. In another example, a monoclonal antibody (83–14 Mab) targeting IR was modified on solid lipids to improve brain-targeted delivery of saquinavir [66] (Fig. 6C). However, brain-targeted delivery systems targeting the insulin receptor remain understudied, likely due to the high cost of using insulin or insulin analogs as ligands and the risk of degradation or inactivation before reaching target tissues.

##### Folate receptor

The folate receptor (FR) family consists of three or four isoforms: FRα, FRβ, FRγ and FRδ, encoded by the FOLR1, FOLR2, FOLR3 and FOLR4 genes, respectively. FRα is highly expressed in the BBB and tumor tissues, which is considered as an ideal target for tumor-targeted therapy [67]. Nanosystems targeting folate receptors have been developed to enhance drug delivery across the BBB and improve the specific recognition and treatment of brain lesion cells. Folic acid can be conjugated with drugs to form folic acid-drug conjugates, which enter tumor cells via FRα-mediated endocytosis, enabling targeted tumor treatment. For example, exosomes conjugated with folic acid and loaded with TMZ and quercetin traversed the BBB via FR, demonstrating inhibitory effects on GBM [68] (Fig. 6D). In addition, folate-modified hollow titanium dioxide (HT) nanorods (HT-FA) were used for targeted delivery of TMZ (HT-TMZ-FA), effectively enhancing glioma targeting and prolonging TMZ's circulation time [69]. Surface modification of polyethylene glycol (PEG)-polycaprolactone-carrying nanoparticles with FRα resulted in higher folic acid accumulation in the brain, showing potential application for treating central system disorders [70]. Despite the promise of folate receptor-targeted delivery systems, variations in folate receptor expression levels across different brain diseases may impact targeting efficiency.

##### N-acetylcholine receptor(nAChR)

Nicotinic acetylcholine receptors (nAChRs) are widely expressed in brain tissue, including brain capillary endothelial cells [71]. RVG29, a peptide that specifically binds to nAChR, can effectively cross the BBB. For example, RVG29-modified PEG hyperbranched poly-L-lysine (DGLs) loaded with non-coding RNA (pshBACE1-AS) has been used to treat AD. It ameliorated AD symptoms by down-regulating the expression of β-site amyloid precursor protein cleaving enzyme (BACE1) [72] (Fig. 6E).

##### Other receptors

In addition to the aforementioned receptors, scavenger receptor BI (SR-BI), diphtheria toxin receptor (DTR) and bradykinin B2 receptor (B2R) are also expressed at the BBB. SR-BI is the main receptor for high-density lipoprotein (HDL). In brain-targeted delivery, SR-BI may facilitate the transport of drugs or therapeutic molecules to the brain through interactions with specific ligands [73]. DTR, a cell surface receptor, allows diphtheria toxin (DT) to enter cells and exert toxic effects. In brain-targeted delivery, DTR can serve as a potential target for delivering drugs or therapeutic molecules to specific brain cells by conjugating them with DT or its derivatives [74]. B2R, a member of the G protein-coupled receptor superfamily, is involved in various biological processes by activating downstream signaling pathways mainly through binding to bradykinin. In brain-targeted delivery, B2R may facilitate the transport of drugs or therapeutic molecules to the brain via interactions with bradykinin or its analogs [75].

Among receptor-mediated transport methods, antibody–drug conjugates (ADCs) and ligand-targeted nanoparticles currently show the satisfactory therapeutic promise due to their clinical translatability and versatility. ADCs (HER2-targeting trastuzumab emtansine) combine antibody specificity with potent payload delivery, evidenced by FDA approvals in oncology, though challenges like off-target toxicity persist. Ligand-decorated nanoparticles (folate/transferrin-functionalized systems) exploit overexpressed receptors for targeted drug/gene delivery while leveraging the EPR effect, but their efficacy is limited by immune clearance and manufacturing complexity. For CNS diseases, receptor-mediated transcytosis (TfR-targeting bispecific antibodies) stands out for overcoming the BBB, yet payload constraints and safety risks require further optimization.

#### Transporter-mediated transportation through BBB

**Fig. 7:**
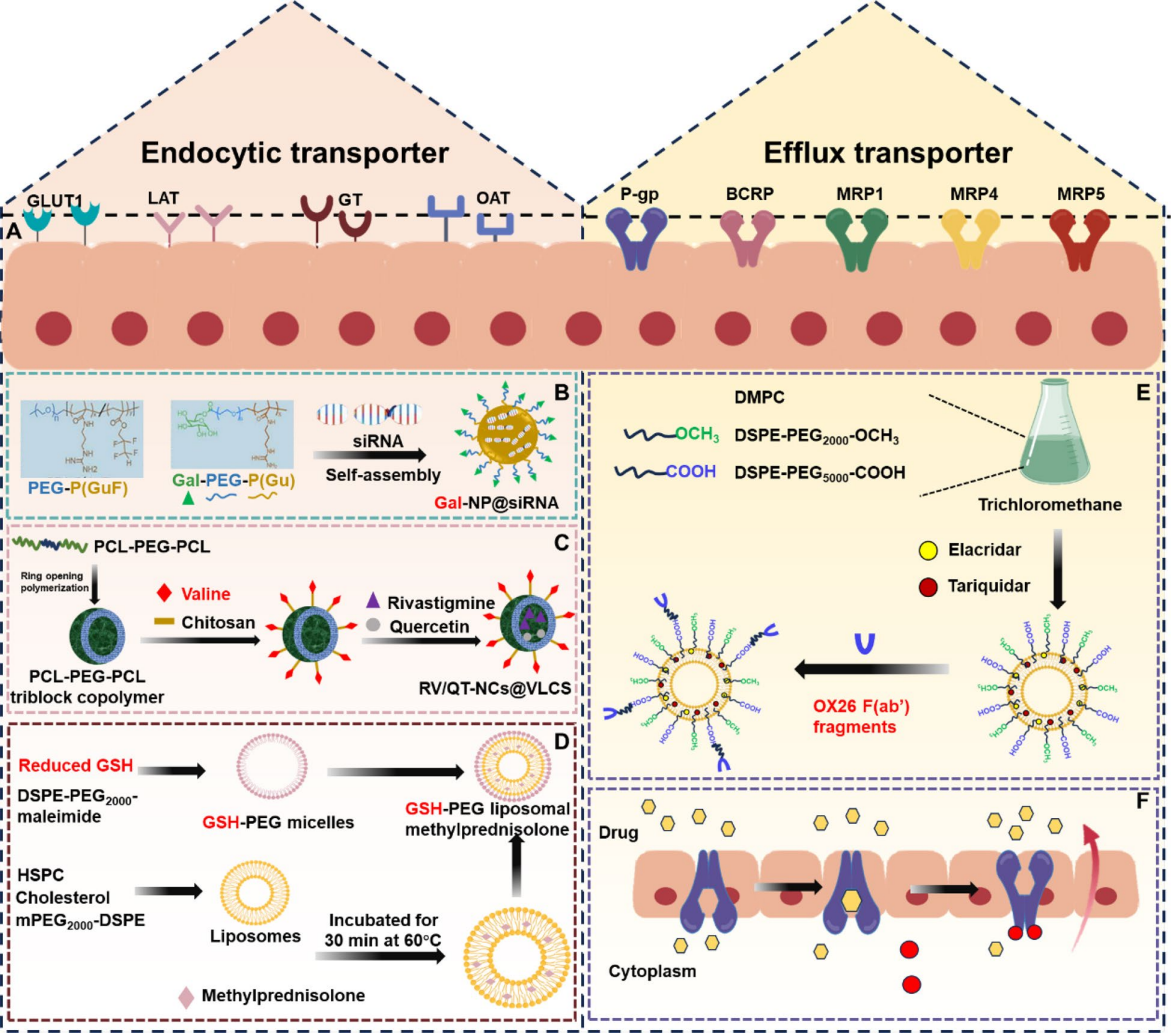
Transporters-mediated strategies for crossing the BBB. **A** Endocytic transporters (GLUT1, LAT, GT, OAT) and efflux transporters (P-gp, BCRP, MRPs) are expressed at the BBB. **B** Galactose (Gal)-modified siRNA nanomedicines penetrate the BBB by GLUT1-mediated transport. **C** Valine-conjugated polymeric nanocarriers loaded with rivastigmine and quercetin cross the BBB via LAT1. **D** PEG liposomes-conjugated GSH encapsulating methylprednisolone crosses the BBB via GT. **E** OX26 F(ab') fragments-conjugated PEGylated liposomes loaded with elacridar and tariquidar improved the brain uptake via inhibiting the P-gp activity. **F** Schematic of the efflux transporters removing drugs from the brain

The brain-targeted delivery strategy mediated by transporters of the BBB exploits the transport proteins specifically expressed on the BBB (such as the GLUT1 transporter, the L-type amino acid transporter, etc.) to achieve efficient transmembrane drug delivery. This strategy improves the distribution of drugs in brain tissue and therapeutic efficacy by designing drugs as analogues of the natural substrates of transporters or conjugating them with the substrates, leveraging the active transport mechanism of transporters to overcome the restrictions of the BBB. In this section, we summarized common transporters-mediated strategies, including glucose transporters, amino acid transporters, glutathione transporters and efflux transporters (Fig. 7A).

##### Glucose transporter protein 1 (GLUT1)

SLC2A1, commonly referred to as glucose transporter protein 1 (GLUT1), is highly expressed in brain endothelial cells. Leveraging the properties of GLUT1, researchers have designed nanocarriers capable of binding to GLUT1, enabling them to cross the BBB via GLUT1-mediated transport. For instance, a glycosylated “triple-interacting” stabilized polymeric siRNA nanomedicine (Gal-NP@siRNA) was developed to efficiently penetrate the BBB through GLUT1-mediated transport (Fig. 7B). It ensured that the siRNA reduced BACE1 expression and modified the relevant pathways, and eventually restored cognitive ability in AD mice [76]. In another study, researchers developed a nanoparticle capable of overcoming multiple complex barriers to delivery into the brain by grafting α-mannopyranoside onto the terminal end of PEG. This modification enabled dual targeting of GLUT1 on both intestinal epithelial cells and brain endothelial cells, facilitating multi-targeted treatment of AD [77].

##### L-type amino acid transporters(LAT)

The L-type amino acid transporter (LAT) is a transmembrane protein responsible for transporting large neutral amino acids. It is highly expressed at the BBB and in tumor cells, making LAT an ideal target for drug delivery to the CNS and various cancers [78]. A common strategy for brain delivery using LAT1 involves designing prodrugs by linking drug fragments to transporter-recognizable substrates. For example, researchers designed and synthesized a LAT1-mediated transport of nipecotic acid precursor drug, which improved BBB penetration and demonstrated antiepileptic activity [79]. Another study reported that valine-conjugated chitosan (VLCS) modified the surface of nanocarriers based on polycaprolactone-polyethylene glycol-polycaprolactone (PCL-PEG-PCL) triblock copolymers. It achieved brain-targeted co-delivery of rosuvastatin and quercetin in AD model, enhancing therapeutic efficacy through LAT1-mediated transport [80] **(**Fig. 7C**).**

##### Glutathione transporter

Glutathione (GSH) is an endogenous tripeptide with antioxidant properties that enters the brain through specific binding to glutathione transporters on the BBB. These transporters are enriched at the BBB and have been effectively utilized in the design of brain-targeted prodrugs and brain drug delivery systems [81]. For example, GSH was combined with magnetic nanoparticles as a BBB shuttle peptide for brain delivery of paclitaxel (PTX). Studies demonstrated that this combination promoted PTX internalization in the brain [82]. In another study, methylprednisolone was delivered by GSH-conjugated PEGylated liposomes (Fig. 7D). Plasma circulation and brain absorption were significantly increased, which improved its efficacy in the treatment of acute experimental autoimmune encephalomyelitis [83].

##### Efflux transporter

BBB endothelial cells express a variety of transporters, including influx and efflux transporters. Efflux transporters, primarily ATP-binding cassette (ABC) transporters and solute carrier (SLC) family members, play a critical role in maintaining the selective permeability of the BBB [84]. P-gp, breast cancer resistance protein (BCRP) and multidrug resistance-associated protein (MRP) are highly expressed on the BBB. These transporters actively pump drugs out of the brain, reducing drug concentrations and compromising therapeutic efficacy [85]. In recent years, researchers have developed a variety of strategies to overcome the efflux transporter [9]. For example, gene therapy has been used to reduce P-gp expression at the BBB, and RNA interference (RNAi) technology has been employed to silence the P-gp gene [86]. In addition, encapsulating drugs in specific nanocarriers can simultaneously facilitate drug delivery and inhibit efflux transporters. One study reported the use of polyethylene glycol-modified liposomes to deliver elacridar and tariquidar across the BBB while reducing P-gp activity, thereby overcoming P-gp-mediated efflux [87] (Figs. 7E and F).

##### Organic anion transporting polypeptides

Organic anion transporting polypeptides (OATPs) represent a category of membrane transport proteins primarily tasked with the translocation of diverse endogenous and exogenous organic anions across the cellular membrane, facilitating their movement from the extracellular environment into the cells [88]. In stroke research, 3-hydroxy-3-methylglutaryl coenzyme A (HMG-CoA) reductase inhibitors, commonly known as statins, have been shown to improve neurological outcomes post-stroke. This characteristic necessitates translocation across the BBB facilitated by the organic anion-transporting polypeptide (OATP1A4) [89]. Recent research indicated that large organic anions were actively transported into choroid plexus epithelial cells via the apical OATP1A2 transporter (OATP1A5 in murine models), followed by their efflux into the systemic circulation via the basolateral multidrug MRP. Given that OATP1A2 could transport a wide range of endogenous and exogenous compounds, its localization at the blood-cerebrospinal fluid barrier suggested a novel mechanism for eliminating pharmacological agents and neurohormones from the cerebrospinal fluid [90].

##### Other transporters

Monocarboxylic acid transporters (MCTs) are a family of transporter proteins that enable the rapid passage of monocarboxylic acid compounds, such as pyruvate, lactate, ketone bodies, and short-chain fatty acids, across cell membranes [91]. MCT1 expression at the BBB provides a pathway for drug delivery from the blood to the brain. It was shown that 4-phenylbutyric acid, a potential therapeutic agent for neurodegenerative diseases, may cross the BBB via an MCT1-mediated transport mechanism [92]. Carnitine transporters, especially organic cation/carnitine transporter protein 2 (OCTN2), also play an important role at the BBB [93].

Overall, transporter-mediated strategies leverage endogenous carrier systems (such as GLUT1 for glucose, LAT1 for large neutrals) to shuttle therapeutics across the BBB, offering distinct advantages: (1) High specificity through natural substrate-receptor interactions, minimizing off-target effects; (2) Saturable but efficient uptake, enabling dose-dependent transport; (3) Broad applicability to diverse modalities (small molecules, prodrugs, or conjugate-based systems). Notable successes include L-DOPA (via LAT1) for Parkinson’s disease and prodrugs like gabapentin (exploiting LAT1) [94]. However, critical limitations persist: (1) Molecular constraints (size/chemistry must mimic endogenous substrates); (2) Competitive inhibition by physiological substrates reducing efficacy.

#### Tight junction protein

**Fig. 8:**
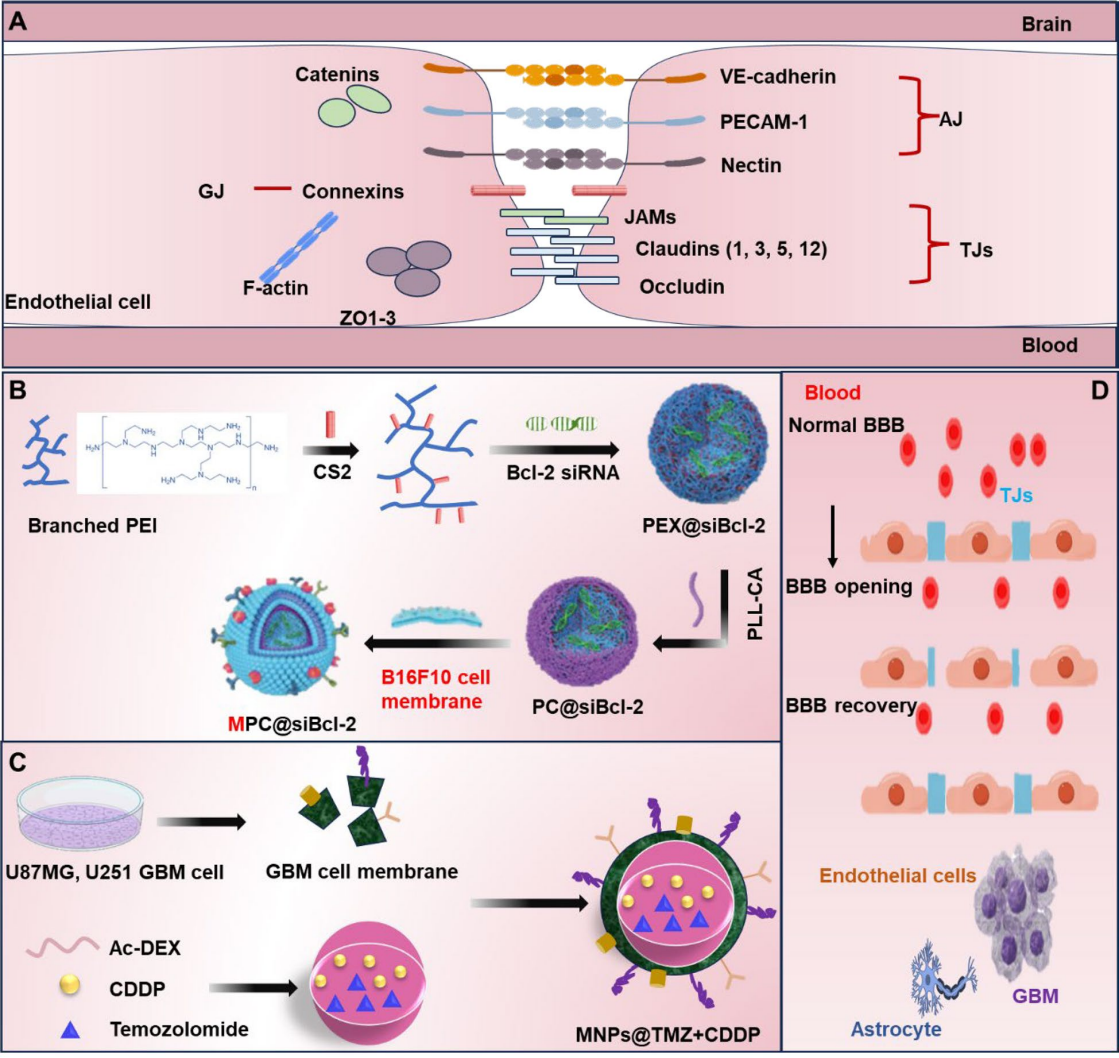
Schematic representation of BBB tight junctions (TJs) and adhesion junctions (AJs) proteins and regulation of vascular endothelial tightness across the BBB. **A** TJs and AJs are expressed at the BBB. **B** Metastatic tumor cell membrane-modified Bcl-2 siRNA (siBcl-2) complexed PEX coated with pH-sensitive charge conversational layer cross the BBB via decreasing the tightness of endothelial cells. **C** New GBM-cell membrane camouflaged nanoparticles (MNPs) co-load TMZ and CDDP (MNPs@TMZ + CDDP) across the BBB by decreasing the tightness of endothelial cells. **D** Schematic diagram of nanoparticles penetrating and recovering the BBB

Tight junction (TJ) proteins of the BBB are predominantly expressed in BMECs. TJ proteins include claudins, occludin, junctional adhesion molecules (JAMs) and cytoplasmic adhesion proteins (ZOs), among others [95] (Fig. 8A). Claudins, particularly Claudin-5, are critical components of intercellular TJs and play a significant role in regulating the integrity and permeability of the BBB [96]. Occludin is a quadruple transmembrane protein expressed mainly in epithelial cells and BMECs, facilitating homophilic junctions and regulating intercellular permeability. JAMs are adhesion molecules, with JAM-A prominently expressed in BMECs[97]. JAM-A modulates lymphocyte infiltration and paracellular transport by establishing TJs through homologous interactions. Cytoplasmic attachment proteins, such as ZO-1, ZO-2, and ZO-3, are essential components of TJs within the BBB. Research has shown that the ablation of ZO-1 leads to the disintegration of TJs, thereby compromising BBB integrity [98].

Targeting TJs proteins to modulate the BBB represents an effective strategy for enhancing drug delivery in the treatment of brain diseases. In brain tumor therapy, a study presents a biomimetic nanomedicine utilizing metastatic tumor cell membrane camouflage. The results indicated that the nanomedicine effectively traversed the BBB by downregulating ZO-1 expression and engaging with endothelial cell adhesion molecules [99] (Fig. 8B). Another recent study developed an innovative biomimetic nanomedicine for GBM treatment, featuring a pH-sensitive polymer core and a shell disguised with GBM cell membranes. This nanomedicine enabled the simultaneous delivery of TMZ and CDDP to brain tumors [100] (Fig. 8C). The reversible modulation of BBB TJs is illustrated in Fig. 8D. In non-tumor brain disorders, alterations in TJs proteins expression are closely associated with disease progression. For instance, ischemic stroke development is linked to enhanced the BBB permeability due to modified TJs proteins [101]. Moreover, research indicated that BBB disruption accelerated AD progression [102]. However, the BBB restricts therapeutic drug delivery to brain tissue, presenting a significant challenge in AD treatment. A new study demonstrated the efficacy and safety of ultrasound-mediated reversible BBB opening combined with aducanumab in AD patients [103]. Therefore, maintaining the integrity of BBB TJs proteins is crucial for preventing and treating neurological disorders. Overall, transient and reversible BBB permeability may offer novel strategies for drug delivery in brain disease management.

#### Adsorption endocytosis-mediated transportation

Adsorptive endocytosis-mediated transport exploits electrostatic interactions between positively charged ligands (such as cationic proteins, cell-penetrating peptides, or nanoparticle surfaces) and negatively charged glycoproteins (such as heparan sulfate proteoglycans) on the BBB endothelial surface [104]. This triggers vesicle formation and internalization, enabling transmembrane delivery of therapeutics without receptor-specific targeting. A recent study developed a dual-modal probe by using dendrimers as carriers loaded with a general control non-repressed protein 5 (GCN5)-targeting small-molecule inhibitor. The probe efficiently crosses the BBB via adsorptive-mediated transcytosis, enabling enhanced preoperative tumor boundary delineation with MRI and intraoperative guidance via fluorescence imaging [105].

#### CRISPR-based strategies for BBB modulation

Recent advances in CRISPR technology have enabled targeted genetic modifications to transiently disrupt the BBB for enhanced therapeutic delivery. One study demonstrated that CRISPR/Cas9-mediated knockout of the ABCB1 transporter in GBM cells significantly enhanced chemotherapy response by overcoming both BBB-mediated and tumor cell-intrinsic drug resistance [106]. Although current research on applying CRISPR/Cas9 technology to modulate BBB permeability is still in its early stages, the technology demonstrates tremendous potential for precisely regulating BBB receptors, transporters, and tight junction proteins as it continues to advance.

### Cellular strategy

Cellular strategy-mediated brain-targeted delivery strategy is an innovative approach that utilizes natural cells or their derived carriers (such as exosomes, cell membrane vesicles) as drug delivery vehicles to efficiently cross the BBB and target drugs to brain tissues. This strategy leverages the inherent cellular chemotaxis, penetrability, and low immunogenicity of these carriers. Currently, extensive research focuses on vectors such as stem cells (such as mesenchymal stem cells), immune cells (such as macrophages, T-cells) and erythrocytes. Additionally, cell-derived exosomes and membrane vesicles have become a research hotspot due to their natural drug-carrying capacity and excellent biocompatibility. This study summarizes the application of various cellular strategies in animal models of brain tumors, neurodegenerative diseases, and ischemic stroke.

#### Exosome

**Fig. 9:**
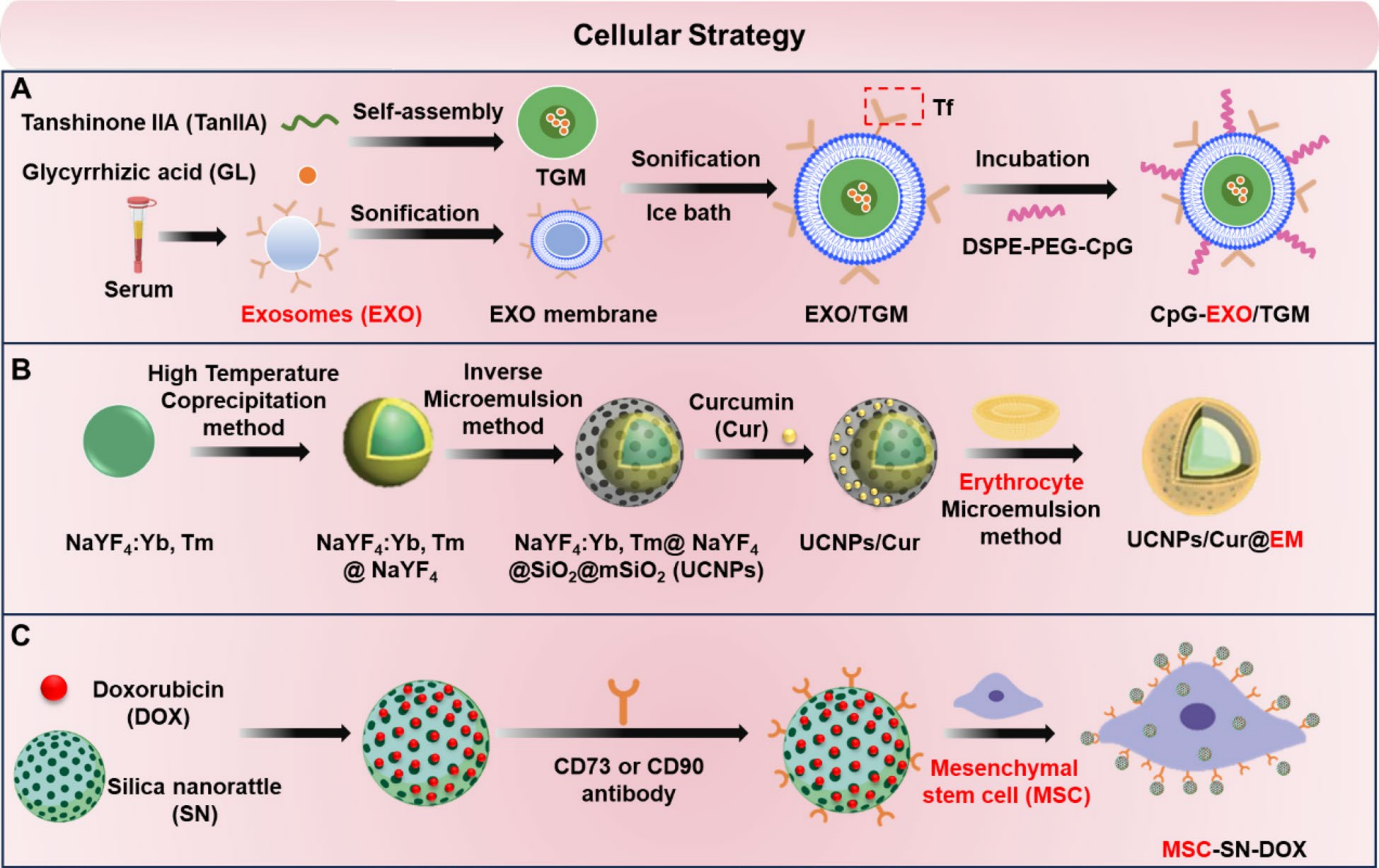
Schematic diagram of cell strategy mediated crossing of the BBB. **A** CpG oligonucleotides-anchored endogenous serum exosomes-coated tanshinone IIA (TanIIA) and glycyrrhizic acid (GL) nanomicelles (CpG-EXO/TGM) cross the BBB by binding the free transferrin. **B** Erythrocyte membrane (EM)-modified mesoporous silica loaded curcumin nanoparticle (UCNP/Cur@EM). **C** Silica nanorattle-doxorubicin-anchored mesenchymal stem cells (MSC-SN-DOX)

Exosomes are small vesicles of cellular origin with good biocompatibility and low immunogenicity. As a new type of natural drug delivery vehicle, it has the property of crossing the BBB [107]. Researchers developed immunoexosome-loaded drug nanoparticles (CpG-EXO/TGM) by self-assembling tanshinone IIA (TanIIA) and glycyrrhizic acid (GL) into TanIIA-GL nanoparticles (TGM). They then utilized serum exosomes as carriers to load TGMs and anchor CpG oligonucleotides onto the exosome membrane for chemo-immunotherapy of GBM across the BBB [108] (Fig. 9A). Additionally, exosomes from M2-type macrophages (M2exo) were utilized to deliver DNase 1 for stroke therapy. These exosomes crossed the BBB via transcytosis and specifically accumulated in ischemic regions. M2exo-derived anti-inflammatory cytokines induced microglial polarization toward the M2 phenotype, exerting neuroprotective effects [109]. Although transcytosis has been identified as the primary pathway for natural exosome translocation across the BBB, the precise molecular mechanisms by which exosomes modulate the BBB remain incompletely understood due to their diverse origins and varying disease contexts. Further investigation is required to elucidate the distinct pathways exosomes employ to traverse the BBB [110].

To enhance the efficacy of exosomes as carriers across the BBB, numerous studies have focused on engineering exosomes and modifying ligands to improve targeting and drug delivery efficiency. For instance, researchers have developed functionalized exosomes with dual targeting of Ang-2 and TAT, effectively targeting both the endothelial surface of the BBB and glioma cells, thereby enhancing the efficacy of in situ glioma therapy [111]. A recent study developed a ROS-responsive bionic nanovesicle through the hybridization of stem cell exosomes and liposomes for the co-delivery of two gene therapy agents: β-secretase 1 (BACE1) siRNA and TREM2 plasmid. Bionic nanovesicles, aided by exosome homing and Ang-2 peptide, efficiently traverse the BBB, enhancing drug accumulation at AD lesions and modulating microglial function to intervene in amyloid β-protein (Aβ) anabolism [112]. Despite their promise as drug delivery vehicles, exosomes face several translational challenges, including limitations in scalable production, inadequate control over in vivo biodistribution and pharmacokinetics.

#### Cell membrane

Cell membrane-modified nanoparticles are crucial for traversing the BBB. Their excellent biocompatibility and low immunogenicity improve circulation time and in vivo stability [113]. A study developed an erythrocyte membrane-modified biomimetic nanoparticle (UCNP/Cur@EM) loaded with curcumin to inhibit Aβ aggregation, reducing Aβ toxicity on neuronal cells and enhancing cognitive and memory functions in AD animal models [114] (Fig. 9B). Notably, P-selectin glycoprotein ligand-1, integrin α4 and macrophage-1 antigen expressed on macrophage membranes played an important role in macrophage penetration of the BBB or targeting of the GBM [115]. Another study developed hollow mesoporous silica nanocarriers encapsulated with death-associated protein kinase 1 inhibitor TC-DAPK6 and rhodamine B, which were coated with macrophage membranes, enabling them to cross the BBB, selectively accumulating at inflammatory foci in epileptic lesions [116].

#### Homing capacity of stem cells

Numerous studies have utilized mesenchymal stem cells (MSCs) as drug carriers for miRNAs, proteins, anti-tumor agents, and other therapeutics to traverse the BBB. In 2011, a study described an innovative tumor-targeting therapy that utilizes bone marrow MSCs as a delivery vehicle in combination with silica nanorattle loaded with DOX. Silica nanorattle loaded with DOX and conjugated with CD73 or CD90 antibodies on the surface were specifically uploaded to MSCs via the antibodies (Fig. 9C). A novel lysogenic adenovirus was engineered using the Tet-on system and delivered into human umbilical cord blood mesenchymal stem cells with IL-24 and endostatin genes to facilitate safe and effective glioma therapy through the modulation of viral replication [117]. Recent studies have introduced a novel PD-L1 and AKT-modified umbilical cord mesenchymal stem cell that enhances neuroplasticity ischemic stroke by improving cell viability and modulating inflammation [118]. Stem cells as drug carriers exhibit high selectivity and efficiency, facilitating tissue repair and regeneration. However, the precise mechanisms of stem cell homing, particularly under pathological conditions, remain inadequately understood. This lack of understanding may impact drug delivery efficacy and precision.

### Physical strategy

#### Focused ultrasound technology and microbubbles

**Fig. 10:**
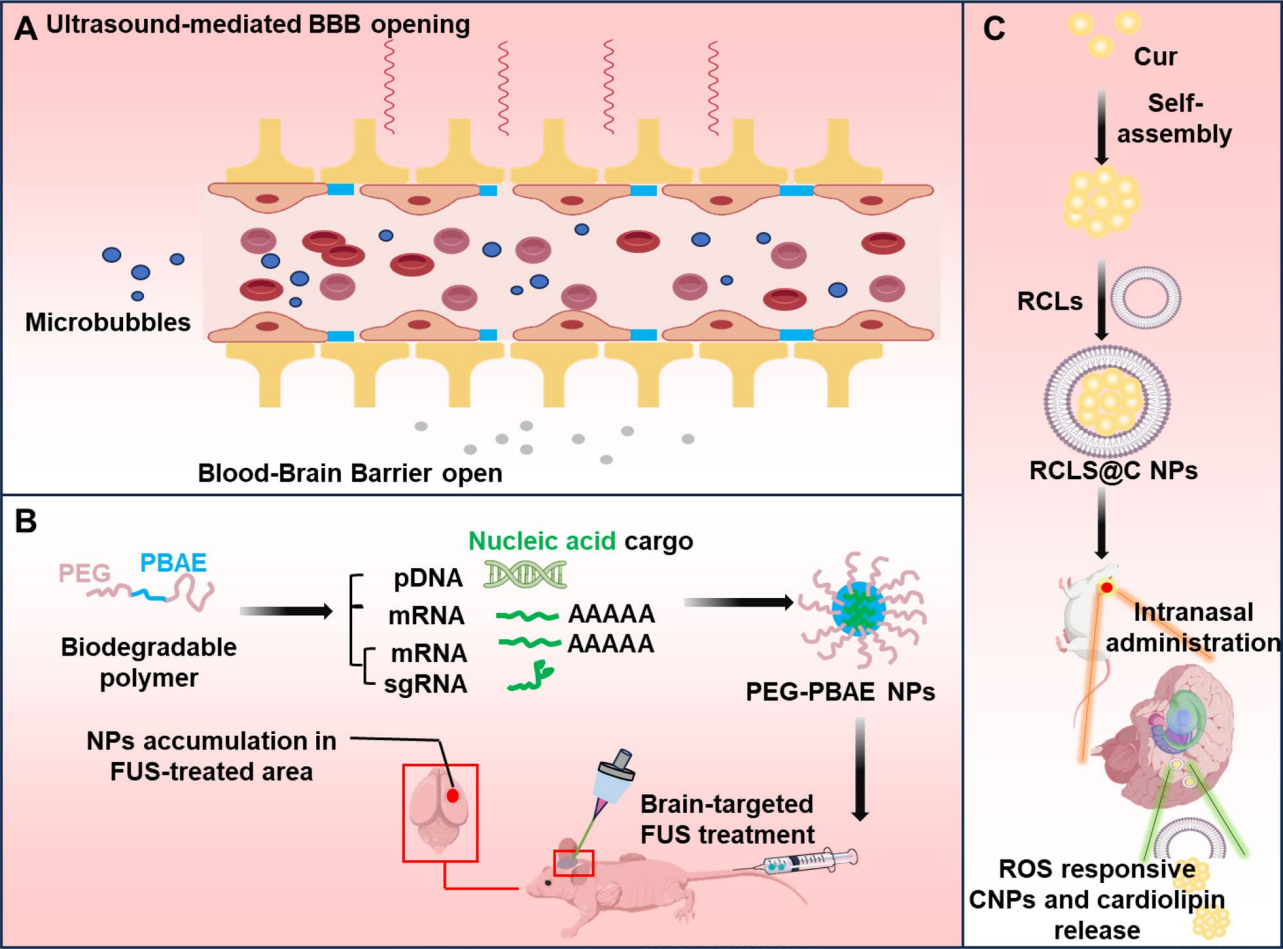
Schematic representation of BBB opening mediated by physical strategies and intranasal administration. **A** Ultrasound-mediated opening of the BBB. **B** FUS-mediated BBB opening and systemic delivery of plasmid DNA and mRNA to brain. **C** Intranasal injection of curcumin nanoparticles (CNPs) loaded responsive cardiolipin liposomes (RCLs) to obtain RCLs@C NPs

Microbubble-assisted FUS is a novel technique for treating brain disorders that utilizes FUS energy to transiently disrupt the BBB, facilitating the delivery of drugs or therapeutic agents into the brain (Fig. 10A). Opening the BBB by FUS technology significantly improved the delivery efficiency of aducanumab in the brains of patients with AD, resulting in lower Aβ levels [119]. Furthermore, researchers are investigating novel methods to anchor microbubbles to cerebral endothelial cells for low-energy ultrasound-mediated drug delivery across the BBB. This method allows microbubbles to accumulate on brain microvessels and even low-energy ultrasound, which poses fewer safety risks compared to conventional FUS and produces a strong cavitation effect that opens the BBB while causing negligible damage to brain tissue [120]. With the development of FUS technology, a recent study proposed a systematic strategy combining FUS to open the BBB with long-circulating biodegradable nanoparticles. Utilizing a biodegradable poly-β-amino ester polymer, the study developed nanoparticles that can effectively encapsulate a diverse array of nucleic acid payloads while exhibiting prolonged circulation and robust serum stability in vivo. Gene editing within astrocytes and neurons in FUS-exposed cerebral regions was accomplished by employing FUS to temporarily permeabilize the BBB, facilitating the passage of nanoparticles across the barrier and their subsequent accumulation in the targeted FUS-treated areas of the brain [121] (Fig. 10B). Furthermore, a research team identified a highly effective two-photon photosensitizer (MeTTh) that demonstrated remarkable aggregation-induced emission characteristics, near-infrared II excitation, and reactive oxygen species generation capabilities. To enhance tumor targeting, transferrin was modified on the surface of MeTTh (MeTTh NPs-Tf) and combined with FUS to open the BBB, enabling MeTTh NPs-Tf to successfully target GBM, which exhibited significant tumor growth inhibition as well as enabling deep brain imaging [122]. Although microbubble-assisted FUS technology holds promise for treating brain disorders, precise control over BBB opening remains a challenge.

FUS-induced BBB opening was found to be accompanied by an acute inflammatory response under high-dose microbubbles conditions [123]. Despite BBB opening after FUS + MBs treatment, no changes in infiltration or migration of immune cells within the tumor were observed, which may indicate that BBB opening does not necessarily trigger significant immune activation under specific conditions [124].

#### Low-intensity pulsed ultrasound (LIPUS)

Low-intensity pulsed ultrasound (LIPUS) comprises periodic mechanical sound waves that traverse cellular and tissue structures, generating vibrations and interactions with negligible thermal consequences. It demonstrates beneficial effects in neuromodulation, such as augmenting neuronal activity, suppressing neuroinflammation and promoting the neural differentiation of stem cells. In contrast to high-intensity FUS, LIFUS allows for precise energy targeting in specific brain regions, facilitating non-invasive interventions for CNS disorders. When combined with intravascular microbubble injections, it can effectively, safely, and reversibly disrupt the BBB [125]. In a clinical study, researchers employed the LIPUS with intravenous microbubbles technique to transiently disrupt the BBB, facilitating the delivery of large molecule drugs, specifically increasing the concentration of albumin-bound PTX in brain tissue [126].

#### Magnetic field-guided delivery

The magnetic targeting strategy exploits magnetic nanoparticles guided by an applied magnetic field to achieve precise drug delivery. A study has prepared a novel brain-targeted delivery platform that combines the magnetic targeting properties of magnetic nanoparticles, drug delivery properties and the BBB penetration ability of Angiopep-2-Lamp2b-modified human MSCs-derived exosomes. Small interfering RNA for GPX4 (siGPX4) was loaded into exosomes using electroporation for synergistic ferroptosis therapy in GBM. Notably, the platform constructed a magnetic helmet for mice by 3D printing, enabling nanomedicines to be enriched in the brain under magnetic localization [127].

### Intranasal administration

Intranasal drug administration represents a non-invasive and efficient method for targeting the CNS. This approach leverages olfactory and trigeminal pathways to circumvent the BBB, facilitating the direct delivery of therapeutic agents to the brain [128]. Research employed nanoprecipitation techniques to formulate PLGA-based polymer nanoparticles encapsulated with edaravone for the purpose of intranasal drug delivery. An in vivo assessment revealed that the intranasal route of administration facilitated superior cerebral uptake compared to caudal venous administration [129]. Recent research has demonstrated that self-assembled curcumin nanoparticle liposomes, when administered intranasally, can influence the polarization of microglial cells in the context of AD. This innovative strategy effectively polarizes microglia by inhibiting Aβ aggregation extracellularly and suppressing inflammation-related pathways intracellularly, thereby offering a promising therapeutic avenue for the treatment of AD [130] (Fig. 10C). The nasal delivery of composite nanoparticles is anticipated to be a more advantageous method of drug administration. This approach offers several benefits, including the ability to efficiently traverse the BBB, improve cerebral bioavailability, minimize adverse drug reactions and specifically target pathologies in the treatment of brain disorders.

## Aroma-opening natural products cross the BBB

In Traditional Chinese Medicine (TCM) theory, orifices serve as vital portals connecting the internal and external body, with their pathological changes reflecting visceral conditions. Aromatic Chinese herbs (containing aromatic components) possess orifice-opening and obstruction-clearing properties. Aromatic compounds, with their pungent and fragrant nature, are known to unblock orifices and facilitate their functioning. Modern research demonstrates these drugs can bidirectionally regulate the BBB. They enhance permeability to facilitate drug penetration while also reducing permeability to protect barrier function. Recent studies have increasingly examined a number of aromatic natural compounds exert the ability to penetrate the BBB and exert therapeutic effects in the CNS. Certain aromatic Chinese herbs such as borneol, *moschus*, *acori tatarinowii rhizome*, styrax and benzoinum have the effect of 'introducing drugs upward' and promote other drugs to cross the BBB [131]. Aromatic compounds present in these aromatic Chinese herb’s extracts can traverse the BBB, either by regulating its permeability or leveraging their inherent lipophilicity. Co-administration of these compounds with other pharmacological agents can promote their translocation across the BBB, thereby augmenting their distribution and therapeutic efficacy within cerebral tissue. The mechanisms by which natural products mediate BBB crossing, along with their structural characteristics, are summarized in Table 3.

**Table 3:** Natural products cross the blood–brain barrier, action mechanism and structure

Natural product	The mechanism of enhanced Blood–brain barrier permeability	Structure	Reference
Menthol	Down-regulate the expressions of ZO-1 and Claudin-5 in brain microvascular endothelial cells, inhibit P-gp activity		[139]
Borneol	Down-regulate the expressions of ZO-1, Claudin-5, occludin in brain microvascular endothelial cells, inhibit P-gp activity, increase 5-hydroxytryptamine content		[133]
Muscone	Down-regulate the expression of Claudin-5, inhibit P-gp activity		[141]
Ligustilide	Down-regulate the expressions of Claudin-5 and ZO-1		[196]
Senkyunolide A	Down-regulate the expressions of Claudin-5 and ZO-1		[197]
Senkyunolide I	Down-regulate the expressions of Claudin-5 and ZO-1		[198]
Gastrodin	Inhibit P-gp activity		[199]
Taurocholic Acid	Organic anion transporters		[200]
α-asarone	Down-regulate the expressions of ZO-1, Claudin-5, occludin in brain microvascular endothelial cells, inhibit P-gp activity		[144]
β-asarone	Down-regulate the expressions of ZO-1, Claudin-5, occludin in brain microvascular endothelial cells, inhibit P-gp activity		[145]
Gambogic amide	Unclear		[201]
Resveratrol	Unclear		[202]
Quercetin	Unclear		[203]
(-)-Epigallocatechin gallate	Unclear		[204]
Plumbagin	Unclear		[205]

### Borneol

**Fig. 11:**
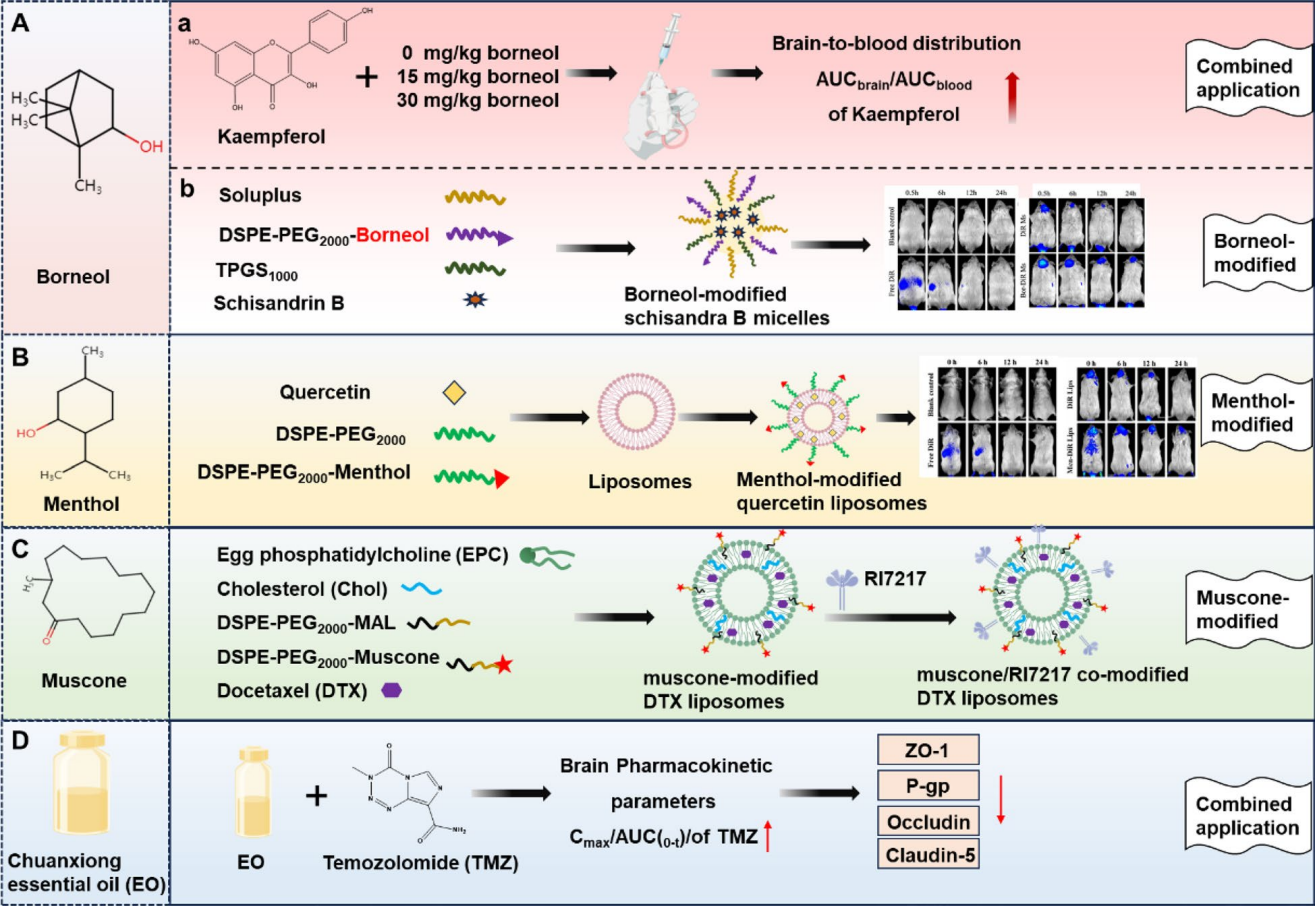
Schematic representation of natural product-mediated crossing of the BBB. **A** Borneol-enhanced the permeability of drugs across the BBB. a) Enhanced brain concentrations of kaempferol in combination with different concentrations of borneol. b) Borneol-modified schisandrin B micelles. Reproduced from Ref. [136] with permission from ACS. **B** Menthol-modified quercetin liposomes. Reproduced from Ref. [24] with permission from ACS. **C** Muscone/RI7217 co-modified DTX liposomes. **D** Chuanxiong essential oil combined application with TMZ and enhanced brain concentrations of TMZ

Borneol is a generally used aromatic drug in TCM, which is a highly fat-soluble bicyclic terpene compound [132]. Numerous studies indicated that borneol inhibited P-gp activity and reversibly modulated TJs proteins expression, thereby enhancing drug permeability across the BBB in neural tissue [133, 134]. Borneol combined with kaempferol enhances its transport across the BBB, boosting bioavailability and brain concentration [135] (Fig. 11A). With advancements in nanotechnology, researchers have explored the study of borneol modified nanomedicines in improving BBB permeability. A recently published investigation developed a borneol-modified schisandrin B micelles utilizing a thin film dispersion technique (Fig. 11A). The targeting efficacy of borneol was assessed through an in vitro BBB model, while the in vivo distribution, circulation duration, and therapeutic effectiveness of the micelles were evaluated in a living organism. The findings indicated that the borneol-modified nanomicelles enhanced drug uptake in bEnd.3 cells and significantly improved the distribution and circulation time of the drug within brain tissue [136]. Additionally, borneol-modified docetaxel plus tetrandrine micelles enhanced drug permeability across the BBB and exhibited improved efficacy against drug-resistant gliomas [137]. Borneol has also been found to have an inverse regulatory effect on BBB permeability, which may be due to differences in the pathologic environment of different diseases. It was found that borneol enhanced BBB permeability and restored its normal function, thereby repairing brain damage and protecting brain tissue, which may be related to inflammatory regulatory mechanisms. The anti-inflammatory and protective effects of borneol can be used to ameliorate and treat ischemic stroke. In addition, when combined with other drugs, borneol accelerate the opening of the BBB, increasing permeability through physiological processes and enhancing drug penetration and distribution in the brain without causing pathological damage to the brain [138]. On the other hand, it may be due to the dose-dependent biphasic effect of borneol that excessive concentrations may reduce permeability or cause barrier tightening [135].

### Menthol

Menthol is a naturally occurring cyclic terpene alcohol with the potential to facilitate drug passage across the BBB. Recent studies indicate that menthol increases BBB permeability, facilitating improved drug delivery to the brain. One study indicated that menthol serves as a drug modifier, facilitating the transport of quercetin across the BBB to the lesion site, thereby increasing its brain concentrations and ensuring effective delivery [24] (Fig. 11B). In another study, menthol modified self-assembled nanoparticles of albendazole (Abz) and nanosilver (MBS-Abz) were developed for glioma-targeted therapy. The results showed menthol enhanced anti-glioma efficacy by promoting internalization and crossing the BBB, facilitating nanoparticle translocation across the endothelial cell monolayer of brain capillaries [139]. Furthermore, menthol-modified casein nanoparticles were synthesized for the encapsulation of the antitumor agent 10-hydroxycamptothecin through the conjugation of menthol with casein, a natural food-derived protein known for its brain-targeting properties. The findings indicated that the menthol-modified nanoparticles exhibited enhanced tumor penetration and improved distribution within brain tumors, significantly extending the median survival of glioma-bearing mice [21]. In summary, the integration of traditional Chinese medicinal approaches with contemporary drug delivery systems offers a novel strategy for the targeted treatment of glioma.

### Muscone

Muscone is known for its ability to induce resuscitation and restore consciousness. Its main pharmacological component, muscone, exhibits a bidirectional regulatory effect on CNS disorders. It can cross the BBB and promote other drugs to enter the brain by regulating the permeability of the BBB and inhibiting the activity of P-gp [140]. Muscone was found to promote the transport of geniposide across the BBB by modulating BBB permeability [141]. In addition, muscone modification enhanced the brain targeting ability of LF-modified liposomes and facilitated the brain delivery of DTX [142] **(**Fig. 11C**)**. This brain-targeted delivery system modified by muscone provides novel ideas for the treatment of CNS diseases.

### Asarone

*β*-asarone is an effective component in the volatile oil of *Acorus tatarinowii* Schott. Some studies have found that asarone can be used as an adjuvant drug in combination with other drugs to improve the brain targeting of drugs [143, 144]. A study demonstrated that *β*-asarone and levodopa elevated dopamine levels in 6-hydroxydopamine-induced Parkinson's disease rats, enhancing therapeutic efficacy by modulating P-gp and TJs proteins at the BBB [145]. Additionally, novel *β*-asarone-functionalized chitosan nanoparticles significantly improved the brain-targeting capability of astragaloside IV (ASI), demonstrating multiple therapeutic benefits in experimental autoimmune encephalomyelitis mice [146]. The optimized nanoformulation not only enhanced ASI accumulation in the CNS but also exhibited dual therapeutic effects: (1) marked reduction of demyelination and (2) promotion of myelin regeneration. These combined actions resulted in superior therapeutic outcomes compared to conventional ASI administration, suggesting *β*-asarone-modified targeted delivery system could represent a promising strategy for treating neuroinflammatory disorders.

### Other ingredients

In addition to the aromatic resuscitation medicines summarized above, other natural products have also been reported to cross the BBB. For example, Chuanxiong rhizome is known as an herb that promotes blood circulation, removes blood stasis and relieves pain. Recent studies have shown that Chuanxiong rhizome and its extracts can increase the BBB permeability by down-regulating the expression of P-gp, Claudin-5 and occludin, thus promoting the passage of drugs across the BBB [147] **(**Fig. 11D**)**. Moreover, certain studies indicated that polyphenols and flavonoids (such as luteolin, apigenin and catechins) could traverse the BBB, potentially related to their lipophilicity [148, 149]. However, the precise mechanisms underlying their action across the BBB remain unclear.

## Toxicity concerns of nanosystems

The increasing utilization of nanosystems for brain-targeted drug delivery has raised significant concerns regarding their long-term safety. While demonstrating promising therapeutic potential, emerging evidence indicates that chronic exposure may lead to neurotoxicity through persistent nanoparticle accumulation. Such as Metallic nanoparticles causing oxidative stress and off-target organ effects such as hepatic fibrosis and renal dysfunction [150]. Small-sized nanoparticles (< 5 nm) are more prone to accumulate in the kidney and trigger glomerular injury [151]. Additionally, a major challenge lies in achieving robust controlled drug release kinetics, as many nanocarriers demonstrate either premature release or incomplete drug liberation. Overall, the complexity of nanosystem increases the difficulty of toxicity assessment because the physicochemical properties of nanomaterials are different from their macroscopic forms, and their toxicity cannot be simply inferred from the feedstock substances. Therefore, despite the great potential of nanosystems for targeted therapy of diseases, the issue of long-term safety and toxicity remains an important field for continued attention.

## Clinical trials

Recent clinical trials have demonstrated significant progress in receptor-targeted therapies for neurological disorders. The bispecific antibody gantenerumab (anti-TfR/anti-Aβ) showed enhanced amyloid plaque reduction in Phase III Alzheimer's trials (NCT03444870 and NCT03443973) though cognitive benefits remain limited [152]. Beyond TfR, LDL receptor-related protein 1 (LRP1)-targeting approaches, such as ANG1005 (a PTX-peptide conjugate), completed Phase II for brain metastases (NCT01967810) [153]. However, challenges remain in receptor saturation and inconsistent therapeutic outcomes across patient populations, driving ongoing optimization of receptor affinity and dosing strategies in current trials.

Notably, recent advances have demonstrated that FUS coupled with microbubble technology holds significant potential for transient BBB disruption, enabling enhanced therapeutic delivery for AD treatment. A recent clinical Phase 1 trial tested a portable neuronavigation-guided FUS system with real-time cavitation monitoring in 6 AD patients. Successful BBB opening was achieved in 5/6 cases (983 ± 626 mm^3^), with cavitation dose correlating strongly with opening volume (R^2^ > 0.9). The procedure (~ 35 min) increased serum AD biomarkers and showed reduced amyloid accumulation in treated brain regions on PET. The system demonstrated safety and feasibility for clinical BBB modulation [154]. Another phase 2 trial evaluated FUS-mediated BBB opening in 8 Alzheimer's patients (mean age 65) across 77 hippocampal, frontal and parietal sites using 220 kHz FUS with microbubbles. MRI demonstrated successful BBB opening with characteristic perivenous contrast accumulation, followed by prolonged venous permeabilization (≤ 1 week) and meningeal enhancement/CSF effusions (≤ 11 days) during closure. All changes resolved spontaneously without serious adverse events [155]. In addition, a clinical validation of receptor-mediated transcytosis using FUS with microbubbles to temporarily disrupt the BBB (NCT04528680), achieving significant drug concentration increases in GBM patients [126]. The application of FUS in CNS treatment has shown promise as a non-invasive and targeted therapeutic modality. Nevertheless, additional large-scale, multicenter clinical trials are warranted to establish its long-term therapeutic outcomes and safety profile, while further technical parameter optimization is needed to expand its applicability across diverse patient cohorts.

## AI-driven modeling for predicting BBB permeability

The computational modeling of molecular capability to cross the BBB is crucial for the development of effective neurotherapeutic drugs. Current computational approaches have evolved from traditional statistical models [156] to classical learning techniques [157], and more recently, large language models (LLM) [158]. From the standpoint of algorithmic accuracy, graph neural network (GNN) [159], flow-based models [160] and transformer [161] contribute unique strengths to predicting BBB permeability (BBBP) when benchmarked against experimental permeability dataset.

However, these models are predominantly developed based on the prediction of molecular physiochemical properties, which is not designated to BBBP specific task and may not fully capture the specific molecular features associated with BBB penetration. Consequently, this raises concerns regarding the interpretability of artificial intelligence (AI) predictions. Furthermore, existing datasets used to train and validation often suffer from reliability issues due to data heterogeneity arising from different experimental methods employed to measure BBBP, as well as insufficient sample sizes in experimental validations [162, 163].

Pharmaceutical formulation is an area under ongoing AI revolution. Computational approach allows researchers to evaluate various scenarios and optimize drug delivery mechanisms without relying heavily on time-consuming trial-and-error experiments [164]. Employment of AI can improve medication formulations by predicting physiochemical properties of drug candidates and by designing and optimizing formulation combination given vast amount of data [165]. By modeling drug delivery systems across multiple scales from molecular interactions to macroscopic behavior employing molecular simulation approaches, AI algorithms, such as artificial neural networks and fuzzy logics, are able to analyze complex relationships between drug properties, formulation components, and physiological factors [166, 167]. These analyses facilitate predictions of drug behavior at each scale, thereby enhancing the understanding of drug delivery mechanisms and aiding in the design of more efficient systems [168]. In the future, model reproducibility and molecular mechanism should be further considered in different formulations targeting brain diseases. In conclusion, while AI has made significant progress in predicting molecular properties such as BBB permeability, ongoing research must address issues related to dataset diversity, model interpretability, and computational resources to further enhance the effectiveness and accessibility of these predictive tools.

## Summary and prospects

The BBB serves as a critical physiological barrier that safeguards the CNS while simultaneously obstructing the passage of numerous therapeutic agents to affected regions of the brain. In this investigation, we comprehensively reviewed the prevailing strategies for targeted delivery across the BBB, alongside the exploration of natural products aimed at modulating BBB permeability. On one hand, nanocarriers engineered through nanotechnology for the encapsulation of chemotherapeutic agents have demonstrated significant promise in the targeted management of neurological disorders. These nanocarriers enhance cerebral accumulation by incorporating specific targeting moieties, such as monoclonal antibodies or particular ligands, enabling them to interact with designated receptors or transporter proteins on the BBB. On the other hand, cell-based delivery methodologies are also under investigation, including the utilization of stem cells or genetically modified immune cells as vehicles to transport therapeutic agents to the brain, leveraging their innate homing capabilities and ability to traverse the BBB. In recent years, the innovative technology of FUS combined with microbubbles has shown great potential for transiently opening the BBB. This approach allows for precise and localized BBB disruption, enabling anticancer, neuroprotective, or gene therapy drugs to penetrate and reach effective concentrations in targeted brain regions, thereby improving therapeutic efficacy. Compared to traditional invasive treatments, this method is minimally or even non-invasive. Despite their potential, these strategies still face challenges such as biocompatibility, carrier stability, large-scale preparation, and the optimization of ultrasound parameters to ensure regional and temporal precision.

Natural products are garnering heightened attention in the investigation of BBB permeability modulation. These compounds are derived from a diverse array of sources, including botanical extracts and microbial metabolites. Certain natural products have demonstrated the capacity to influence BBB permeability through various mechanisms. At the molecular level, some of these compounds can modulate the expression and functionality of TJs proteins, which are critical for preserving the structural integrity of the BBB. For instance, specific aroma-active constituents have been shown to downregulate the expression of TJs proteins, thereby reversibly altering BBB permeability, which is significant relevance in the management of neurological disorders. Additionally, there are natural products that interact with transporter proteins at the BBB, impacting the transmembrane transport of various substances. In recent years, there has been an increasing amount of research on the combined administration of natural products to enhance the BBB permeability, primarily focusing on aromatic Chinese medicines. These TCM ingredients, due to their lipophilic nature, can easily penetrate the BBB and inhibit the activity of P-gp, thereby increasing the concentration of drugs entering the brain. They can also inhibit the expression of Claudin-5 protein, widen the gaps between tightly connected endothelial cells, and increase BBB permeability.

It is noteworthy that the combination of aromatic Chinese medicines with modern formulations to construct novel brain-targeted drug delivery systems has become a promising research direction. For example, drug-carrying liposomes modified with borneol and menthol improve the distribution of drugs in the brain. This combination of the classical theory of Chinese medicine, "channeling upward", with modern nanotechnology not only enriches the scientific connotation of channel ushering drug, but also provides new ideas and methods for the design of advanced brain-targeted drug delivery systems. However, the complexity of traditional Chinese medicine, including the diversity of its composition and mechanisms of action, poses significant research challenges, limiting its development in the application of brain diseases.

Overall, research on BBB-targeted delivery strategies has brought new hope for the treatment of brain diseases. The research on the modulation of BBB permeability by aromatic resuscitation medicine has been gradually deepened, providing a rich resource for the development of novel brain-targeted agents. Therefore, it is important to further explore the modulating effects of aromatic resuscitation medicine on BBB permeability and their underlying molecular mechanisms, including the effects on tight junction proteins and vesicular transport proteins, as well as the modulation of pathological processes such as inflammatory responses and oxidative stress in the brain. Assessing their distribution, metabolism and excretion in the body, as well as possible side effects and toxicity is also crucial. Notably, the application of AI facilitates the revelation of previously unrecognized structure-permeability relationships in these traditional medicines, thereby enabling more systematic exploration of their BBB-modulating potential. On this basis, aromatic resuscitation medicines as nanocarriers are expected to provide a more effective means for the treatment of CNS diseases in the future.

## Data Availability

No datasets were generated or analysed during the current study.
